# The Specific Inhibition of SOD1 Selectively Promotes Apoptosis of Cancer Cells via Regulation of the ROS Signaling Network

**DOI:** 10.1155/2019/9706792

**Published:** 2019-02-18

**Authors:** Xiang Li, Yuanyuan Chen, Jidong Zhao, Jiayuan Shi, Mingfang Wang, Shuang Qiu, Yinghui Hu, Yulin Xu, Yanfang Cui, Chunrong Liu, Changlin Liu

**Affiliations:** Key Laboratory of Pesticide and Chemical Biology, Ministry of Education, School of Chemistry, Central China Normal University, Wuhan, 430079 Hubei, China

## Abstract

Multiple signaling pathways including ERK, PI3K-Akt, and NF-*κ*B, which are essential for onset and development of cancer, can be activated by intracellularly sustained high levels of H_2_O_2_ provided by elevated activity and expression of copper/zinc superoxide dismutase (SOD1) that catalyzes the dismutation of O_2_^•−^ into H_2_O_2_. Here, tests performed by the utilization of our designed specific SOD1 inhibitor LD100 on cancer and normal cells reveal that the signaling pathways and their crosstalk to support cancer cell growth are repressed, but the signaling pathways to promote cancer cell cycle arrest and apoptosis are stimulated by specific SOD1 inhibition-mediated ROS changes. These regulated pathways constitute an ROS signaling network that determines the fate of cancer cells. This ROS signaling network is also regulated in SOD1 knockdown cells. These findings might facilitate disclosure of action mechanisms by copper-chelating anticancer agents and design of SOD1-targeting and ROS signaling pathway-interfering anticancer small molecules.

## 1. Introduction

The signaling roles of the reactive oxygen species (ROS) hydrogen peroxide (H_2_O_2_) and likely superoxide anion (O_2_^•−^) have been established over the past two decades. The signaling pathways regulated by H_2_O_2_ include mitogen-activated protein kinase (MAPK), AMP-activated protein kinase (AMPK), nuclear factor-*κ*B (NF-*κ*B), phosphatidylinositol 3-kinase-Akt (PI3K-Akt), c-Jun N-terminal kinase (JNK), Janus kinase-signal transducers and activator of transcription (JAK-STAT), activator protein-1 (AP-1), hypoxia-inducible factor (HIF), nuclear factor (erythroid-derived 2)-related factor 2 (Nrf2), Ras, and Rac [1–9]. Many of these signaling pathways activated by appropriately high H_2_O_2_ levels promote uncontrollable growth, proliferation, and differentiation of cells, all of which are important hallmarks of tumorigenesis, angiogenesis, and metastasis of cancer cells [10–14], indicating the importance of controlling intracellular H_2_O_2_ levels in cancer treatment.

H_2_O_2_ is generated in mammalian cells mainly via the catalytic dismutation of O_2_^•−^ by copper/zinc and manganese superoxide dismutase (SOD1 and SOD2) [15]. Because SOD1 is an essential antioxidant enzyme that is widely distributed in a cell and provides 80% of total SOD activity [15, 16], the regulatory roles of SOD1 in H_2_O_2_ signaling are increasingly recognized as a new function of SOD1. Multiple signaling pathways including NF-*κ*B, kinases (MAPK, JNK, and Akt), AP-1, and JAK-STAT are activated by the overexpression of SOD1 [17–20]. SOD1 was also found to play important roles in the regulation of tumor necrosis factor-*α*- (TNF-*α*-) induced oxidative stress signaling [18, 21–23] and to serve as a redox sensor to control Rac activation of Nox enzymes in redoxosomes [24]. In addition, SOD1 was observed to act as a metabolic focal point for integrating O_2_, glucose, and ROS to direct energy metabolism [25] and was also suggested to function as a nuclear transcription factor to control general oxidative stress responses [26]. However, whether SOD1 acts as a master regulator of ROS signaling pathways in cancer biology remains elusive.

SOD1 is well known to exist as a key homodimeric metalloenzyme in the antioxidant defense of almost all eukaryotic species. Its each subunit hosts a Cu/Zn site responsible for the catalytic dismutation of O_2_^•−^ into O_2_ and H_2_O_2_ [15, 16]. The small molecules that inhibit intracellular SOD1 activity can be divided into two types: the chelators that target the copper ions essential for the activity of SOD1 and the organic molecules that bind to other sites in SOD1, e.g., 2-methoxyoestradiol [27], 4,5-dichloro-2-m-tolylpyridazin-3(2H)-one [28], and clioquinol [29]. Diethyldithiocarbamate (DDC) and tetrathiomolybdate (TM) are a pair of the most studied copper-chelating inhibitors of SOD1. Although *in vitro* tests showed that DDC and its derivatives inhibit SOD1 at more than millimolar concentrations [30–32], whether they can specifically inactivate SOD1 or not remains elusive. TM and its derivative ATN-224 were found to efficiently inhibit SOD1 via chelating copper in SOD1 [33–35]. Because ATN-224 was observed to suppress cancer cell growth and angiogenesis, it has been tested in phase I–III clinical studies as an anticancer drug [33–39]. ATN-224's anticancer activity is attributed to the inhibition of the growth factor-mediated ERK1/2 phosphorylation indispensable to growth factor signaling because of the SOD1 inhibition-mediated reduction of intracellular H_2_O_2_ levels [40]. However, the inhibitors of SOD1 also inactivate many copper proteins and enzymes including cytochrome c oxidase and ceruloplasmin [41]. Moreover, the copper trafficking essential for normal cellular functions is blocked by the formation of a TM-Cu cluster with the copper chaperone Atox1 [42], although the inhibition of copper trafficking by a small molecule can significantly attenuate cancer cell proliferation [43]. These observations indicate that lack of specific SOD1 inhibitors is a hindrance that needs to be overcome in the exploration of the specific interruption of H_2_O_2_ signaling. Based on the active site structure and catalytic mechanism of SOD1, we designed an efficient copper-chelating and specific SOD1 inhibitor, LD100 [44]. Cell experiments indicated that it did not impact the activity of other copper proteins and enzymes, and its IC_50_ reaches at a nanomolar scale in the inhibition of intracellular SOD1 activity.

The specific SOD1 inhibition-mediated suppression of ROS signaling pathways might trigger cancer cell apoptosis, because the sustained maintenance of highly intracellular H_2_O_2_ levels provided by upregulated expression and activity of SOD1 support the activation of ROS signaling pathways [45–48], resulting in tumorigenesis [48–51]. To verify whether SOD1 inhibition can selectively kill cancer cells and explore the related mechanisms, global mRNA sequencing on cancer and normal cells and other biochemical examinations were performed here. Our findings reveal that the LD100-mediated specific SOD1 inhibition selectively kills cancer cells via regulation of the ROS signaling network that is comprised of signaling pathways to support growth and to promote cycle arrest and apoptosis of cancer cells. Moreover, SOD1 is found to locate at the master hub in the ROS signaling network. Therefore, specific SOD1 inhibition should become a potential anticancer method.

## 2. Materials and Methods

### 2.1. Chemicals and Materials

HRP-conjugated goat anti-mouse IgG (H+L) polyclonal antibody (Cat# ab6789; RRID:AB_955439), HRP-conjugated ganti-rabbit IgG (H+L) polyclonal antibody (Cat# ab6721; RRID:AB_955447), mouse monoclonal anti-beta-actin (Cat# ab8226; RRID:AB_306371), mouse monoclonal anti-caspase-3 (Cat# ab208161), mouse monoclonal anti-ERK1+ERK2 (Cat# ab54230; RRID:AB_2139967), mouse monoclonal anti-PI 3 kinase p85 alpha (Cat# ab86714; RRID:AB_1951326), rabbit monoclonal anti-active caspase-3 (Cat# ab32042; RRID:AB_725947), rabbit monoclonal anti-AKT1 (Cat# ab32505; RRID:AB_722681), rabbit monoclonal anti-AKT1 (phospho S473) (Cat# ab81283; RRID:AB_2224551), rabbit monoclonal anti-Bcl-2 (Cat# ab32124; RRID:AB_725644), rabbit monoclonal anti-cleaved PARP1 (Cat# ab32064; RRID:AB_777102), rabbit monoclonal anti-Erk1 (pT202/pY204)+Erk2 (pT185/pY187) (Cat# ab76299; RRID:AB_1523577), rabbit monoclonal anti-IKB alpha (Cat# ab32518; RRID:AB_733068), rabbit monoclonal anti-IKB alpha (phospho S36) (Cat# ab133462), rabbit monoclonal anti-NF-*κ*B p65 (Cat# ab76311; RRID:AB_2179019), rabbit monoclonal anti-NF-*κ*B p65 (phospho S536) (Cat# ab76302; RRID:AB_1524028), rabbit monoclonal anti-p53 (Cat# ab26; RRID:AB_303198), rabbit monoclonal anti-PARP1 (Cat# ab32138; RRID:AB_777101), rabbit monoclonal anti-superoxide dismutase 1 (Cat# ab51254; RRID:AB_882757), rabbit polyclonal anti-PI 3 kinase p85 alpha (phospho Y607) (Cat# ab182651), BAY117082 (Cat# ab141228), LY294002 (Cat# ab120243), U0126 (Cat# ab120241), Apoptotic DNA Ladder Detection Kit (Cat# ab66090), and Annexin V-FITC Apoptosis Staining/Detection Kit (Cat# ab14085) were purchased from Abcam. Rabbit polyclonal anti-caspase-9 (Cat# 9502; RRID:AB_2068621) and rabbit polyclonal anti-cleaved caspase-9 (Asp353) (Cat# 9509; RRID:AB_2073476) were purchased from Cell Signaling Technology. Rabbit polyclonal anti-CDKN1A (Ab-145) (Cat# SAB4300419), rabbit polyclonal anti-p19 (N-terminal) (Cat# SAB1306548), dihydrorhodamine 123 (Cat# D1054), N^G^-methyl-L-arginine acetate salt (L-NMMA) (Cat# M7033), Rhodamine 123 (Cat# R8004), and propidium iodide solution (Cat# P4864) were purchased from Sigma-Aldrich. PMSF protease inhibitor (Cat# 36978), BCA Protein Assay Kit (Cat# 23225), DAB Substrate Kit (Cat# 34002), Prestained Protein Ladder (Cat# 26616), and TBS Tween buffer (Cat# 28360) were purchased from Thermo Fisher. RNase A (Cat# 12091021), TRIzol (Cat# 15596026), UltraPure Low Melting Point Agarose (Cat# 16520050), and Lipofectamine RNAiMAX Transfection Reagent (Cat# 13778075) were purchased from Invitrogen. Thermolabile USER II Enzyme (Cat# M5508S) and NEBNext Ultra RNA Library Prep Kit for Illumina (Cat# E7530S) were purchased from New England Biolabs. FastStart Essential DNA Green Master (Cat# 06402712001), High Pure RNA Isolation Kit (Cat# 11828665001), and Transcriptor First Strand cDNA Synthesis Kit (Cat# 04897030001) were purchased from Roche. Validated siRNA of SOD1 (Cat# AM51331) were purchased from Ambition.

### 2.2. Cell Lines

Cell lines HeLa and RWPE-1 were obtained from China Center for Type Culture Collection (CCTCC), and DU145 was obtained from American Type Culture Collection (ATCC). All cell lines were maintained at 37°C in a saturating humidity atmosphere containing 5% CO_2_. DU145 and HeLa cells were cultured in DMEM medium (Gibco, C11995500BT) supplemented with 1% penicillin/streptomycin (Gibco, 15140122) and 10% FBS (Gibco, 26140079). RWPE-1 cells were cultured in Defined Keratinocyte-SFM (K-SFM) medium (Gibco, 10785012) supplemented with 1% penicillin/streptomycin and 2‰ Defined Keratinocyte-SFM Growth Supplement (Gibco, 10784015).

### 2.3. LD100 Treatment of Cells

DU145, RWPE-1, and HeLa cells were seeded in a 6-well plate (for general experiments) or 25 cm^2^ cell culture flask (for mRNA sequencing) at a density of 2 × 10^5^ cells/well or 1 × 10^6^ cells/flask in completed DMEM (DU145, HeLa) and K-SFM (RWPE-1) culture medium. After washing cells with D-PBS (RWPE-1) or PBS (DU145 and HeLa), corresponding medium mixed with or without LD100 was incubated with cells for 24 h (except for these time-dependent experiments). Finally, these cells were used to further investigation.

### 2.4. Assays of SOD1 Activity

The cells were lysed using lysis buffer (Thermo Scientific, 23225) supplemented with the PMSF protease inhibitor (Thermo Scientific, 36978). The lysates were rotated in a culturing shaking incubator at 4°C for 30 min and cleared by centrifugation at 12,000 ×g at 4°C for 10 min. After detection of the protein concentration by Pierce BCA Protein Assay Kit (Thermo Scientific, 23225), lysates of same protein content were used to detect the activity of SOD1. SOD1 activity was determined using HT Superoxide Dismutase Assay Kit (Trevigen, 7501-500-K). The activity of SOD1 was determined by measuring the inhibition of tetrazolium salt (WST-1) reduction, which produced a formazan dye upon reduction with a superoxide anion.

### 2.5. Measurements of Intracellular H_2_O_2_ and O_2_^•−^ Levels

For H_2_O_2_ measurements, cells were incubated with final concentration of 5 *μ*M 2′,7′-dichlorofluorescein diacetate (DCFH-DA) (Sigma, 287810) for 20 min at 37°C in the dark. For O_2_^•−^ measurements, cells were incubated with the final concentration of 5 *μ*M dihydroethidium (DHE) (Sigma, 37291) for 20 min at 37°C in the dark. Then, the cells were washed with PBS (DU145) or D-PBS (RWPE-1) three times and resuspended in 200 *μ*L PBS (DU145) or D-PBS (RWPE-1). After subtracting the background fluorescence of LD100, the fluorescence intensity of DHE was obtained. Flow cytometry (BD, Accuri™ C6) was used for all the intracellular fluorescence assays.

### 2.6. Gene Silencing by siRNA Transfection

The validated siRNA of SOD1 was purchased from commercial supplier (Ambion, AM51331). Briefly, when HeLa cells were seeded to be 40%-50% confluent, they were transfected with 90 pmol siRNA in the presence of 20 *μ*L Lipofectamine RNAiMAX (Invitrogen, 13778075) in 4 mL completed DMEM with 600 *μ*L Opti-MEM (Gibco, 31985070) at 37°C for 24 h, according to the manufacturer's instructions. In the next day, cells were transfected again under the same condition to obtain better transfection effect.

### 2.7. Generation and Analysis of mRNA Sequencing Data

HeLa cells (designated as C), LD100-treated HeLa cells (L), SOD1 knockdown HeLa cells (S), DU145 cells (DC), LD100-treated DU145 cells (DL), RWPE-1 cells (RC), and LD100-treated RWPE-1 cells (RL) were used to construct RNA sequencing. Total RNA extraction was performed using TRIzol (Invitrogen, 15596026). Then, 3 *μ*g RNA per sample was used as input material, and sequencing libraries were generated by NEBNext Ultra RNA Library Prep Kit for Illumina (New England Biolabs, E7530S) according to the manufacturer's instructions. Briefly, after purifying mRNA from total RNA by poly-T oligo-attached magnetic beads, fragmentation of mRNA was carried out at elevated temperature in NEBNext First Strand Synthesis Reaction Buffer (5x). Random hexamer primer and M-MuLV Reverse Transcriptase (RNase H^−^) were applied to the synthesis of first-strand cDNA, and DNA polymerase I and RNase H were applied to the synthesis of second-strand cDNA. Remaining overhangs were converted into blunt ends by exonuclease and polymerase. After adenylation of 3′ ends, NEBNext Adaptor with hairpin loop structure's ligation was carried out to prepare for hybridization. The library fragments were purified by the AMPure XP system (Beckman Coulter) to select the cDNA fragments of 150~200 bp. The size-selected adaptor-ligated cDNA was mixed with 3 *μ*L Thermolabile USER II Enzyme (New England Biolabs, M5508S) at 37°C for 15 min followed by 5 min at 95°C. Finally, amplification of the cDNA by PCR was performed, and the PCR products were purified by the AMPure XP system. The clustering of the index-coded samples was performed on a cBot Cluster Generation System using TruSeq PE Cluster Kit v3-cBot-HS (Illumina). At last, the sequencing libraries were sequenced on an Illumina HiSeq platform, and the 125 bp/150 bp paired-end reads are generated. In all cases, three biological replicates were analyzed.

Through in-house Perl scripts, clean reads were obtained from raw reads (FASTQ) by removing low quality, adapter-related, and ploy-N reads. Q20, Q30, and GC content of the clean reads were also calculated. Remaining paired-end clean reads were aligned to the reference genome (GRCh38) using the HISAT aligner [52]. After calculating the reads' numbers mapped to each gene by HTSeq [53], we estimated gene expression levels via fragments per kilobase of transcript per million mapped reads (FPKM). Differential expression analysis of two different groups was performed using the DESeq R package [54], which was based on a model of negative binomial distribution. The resulting *P* values were adjusted by Benjamini and Hochberg's approach to control the false discovery rate. When the adjusted *P* values of genes were less than 0.05, they were assigned as differentially expressed. Based on the FPKM, cluster analysis of differentially expressed genes was performed using ClustVis [55]. KOBAS software was used to test the statistical enrichment of differentially expressed genes in KEGG pathways [56]. GOseq R package was used to perform the Gene Ontology (GO) enrichment analysis of differentially expressed genes [57], and the gene length bias was corrected. GO terms with corrected *P* value less than 0.05 were considered significantly enriched by differential expressed genes.

### 2.8. RT-qPCR

Total RNA extraction was performed using the High Pure RNA Isolation Kit (Roche, 11828665001), and then reverse transcription was performed from 1 *μ*g of total RNA using the Transcriptor First Strand cDNA Synthesis Kit (Roche, 04897030001). Quantitative PCR was performed in a LightCycler 96 (Roche) with FastStart Essential DNA Green Master (Roche, 06402712001) in 20 *μ*L final volume per well. Melt curve analysis was performed to verify the specificity of PCR amplifications. All reactions were performed as triplicate, and then the quantity of mRNA was calculated by the 2^-△△Ct^ method. The following primers were used: SOD1: 5′-AAGGCCGTGTGCGTGCTGAA-3′, 5′-GGCCCACCGTGTTTTCTGGA-3′; *β*-actin: 5′-CCACACCTTCTACAATGAGC-3′, 5′-TGAGGTAGTCAGTCAGGTCC-3′; and p53: 5′-CAGCACATGACGGAGGTTGT-3′, 5′-TCATCCAAATACTCCACACGC-3′.

Quantitative values were obtained from the cycle number (Cq or Ct value). The 2^−ΔΔCt^ method was used to analyze the relative changes in gene expression. Relative quantities were determined as Q_sample_ = 2^−ΔΔCt^, where the ΔCt value was determined by subtracting the average Ct of target gene from the average Ct of the housekeeping gene and ΔΔCt was determined by subtracting the average ΔCt of target gene in experimental groups from the average ΔCt of the same target gene in control groups.

### 2.9. Western Blotting

Cells treated with or without LD100 were constructed as described above. The cells were lysed using lysis buffer (62.5 mM Tris at pH 6.8, 10% glycerol, 2% SDS) supplemented with the PMSF protease inhibitor. The lysates were rotated in a culturing shaking incubator at 4°C for 30 min and cleared by centrifugation at 12,000 ×g at 4°C for 10 min. After detection of the protein concentration by the Pierce BCA Protein Assay Kit, lysates of same protein content were mixed with loading buffer and denatured at 100°C for 5 min. An equal amount of protein and protein ladder (Thermo Scientific, 26616) was loaded on a 10% SDS/PAGE gel. After electrophoresis, proteins were transferred to 0.45 *μ*m PVDF transfer membrane (Thermo Scientific, 88585) and blocked in 1x TBS-Tween (Thermo Scientific, 28360) buffer with 5% BSA. Then, a suitable concentration of primary antibody diluted by 1x TBS-Tween was incubated with the membrane overnight at 4°C. After washing the extra primary antibody, anti-mouse or anti-rabbit horseradish peroxidase-conjugated secondary antibody was added in 1 : 1000 dilution and incubated for 2 h. At last, the Pierce™ DAB Substrate Kit (Thermo Scientific, 34002) was used into the staining of PVDF transfer membrane. Following acquisition, images were imported into ImageJ (NIH) for manual quantitation.

### 2.10. Cell Cycle Assay

Cells treated with or without LD100 were constructed as described above. Then, cells were fixed with 70% EtOH at 4°C for 10 h. After washing with PBS (DU145 and HeLa) or D-PBS (RWPE-1), cells were incubated with PBS or D-PBS/0.25% Triton X-100/20 *μ*g/mL RNase A (Invitrogen, 12091021) at 37°C for 1 h. Then, propidium iodide (Sigma-Aldrich, P4864) was added into the above mixture solution and reached the final concentration 30 *μ*g/mL. Finally, cells were incubated with the PI solution at room temperature for 1 h, and the fluorescence intensity was collected by flow cytometry (BD, Accuri™ C6).

### 2.11. Cell Apoptosis Assay

FITC-conjugated annexin-V assays were used to assess cell apoptosis. Following the manufacturer's instructions of the Annexin V-FITC Apoptosis Detection Kit (Abcam, ab14085), cells were washed by cold PBS (DU145 and HeLa) or D-PBS (RWPE-1) and resuspended in 500 *μ*L of 1x binding buffer. Then, 5 *μ*L of Annexin V-FITC and 5 *μ*L of propidium iodide were added into the binding buffer and incubated at room temperature for 5 min in the dark, and the stained cells were analyzed by flow cytometry.

### 2.12. Mitochondrial Membrane Permeability Assay

Under normal conditions, rhodamine 123 (Rh123) partitioned into polarized mitochondria and self-quenched. When cell apoptosis increased, Rh123 leaked out of the mitochondria into the cytoplasm where it dequenched and fluoresced strongly. Therefore, mitochondrial membrane potential was analyzed by Rh123 (Sigma-Aldrich, R8004). Cells were treated with the respective treatment and incubated with 0.2 *μ*M Rh123 in culture medium for 30 min at 37°C. Then, cells were washed three times with culture medium and resuspended in PBS (DU145 and HeLa) or D-PBS (RWPE-1). The fluorescence intensity of Rh123 was collected by flow cytometry.

### 2.13. DNA Fragmentation Assay

After treating cells with or without LD100 as described above, samples containing about 1 × 10^6^ cells were harvested. The Apoptotic DNA Ladder Detection Kit (Abcam, ab66090) was used to reveal the DNA fragmentation induced by LD100. Briefly, cells were lysed in 35 *μ*L TE lysis buffer, and then 5 *μ*L Enzyme A Solution was added into the lysate and incubated at 37°C for 10 min. Next, 5 *μ*L Enzyme B Solution was added into each sample and incubated at 50°C for 12 h. Then, 5 *μ*L ammonium acetate solution and 50 *μ*L isopropanol (Sigma-Aldrich, I9516) were added into each sample and mixed well. After keeping samples at -20°C for 10 min, the samples were centrifuged 10 minutes at 12,000 ×g to precipitate DNA. Next, the DNA was washed with 0.5 mL 70% ethanol; trace ethanol was removed, and samples were air dried for 10 minutes at room temperature. After dissolving the DNA in 30 *μ*L DNA suspension buffer, the samples were analyzed by agarose gel electrophoresis.

### 2.14. Assay of Peroxynitrite Formation

HeLa cells were seeded in a 6-well plate at a density of 2 × 10^5^ cells/well in completed DMEM culture medium. Next, cells were washed with PBS, and serum-free DMEM medium mixed with LD100 (0, 50, and 100 *μ*M) were incubated with the cells. After 22 h, L-NMMA (0, 1 mM) was added into these samples incubating for another 2 h. Then, the cells were washed three times using PBS and incubated with the peroxynitrite fluorescent probe dihydrorhodamine 123 (DHR123) (Sigma-Aldrich, D1054) in fresh DMEM medium for 1 h in the dark at 37°C. After washing cells three times using PBS, the fluorescence of samples was visualized using the inversion fluorescence microscope Leica DMLB with the Leica filter I3, a filter excitation BP 450–490 nm, FT 510 nm, and emission LP 515 nm. Besides, the samples were further analyzed using flow cytometry at the same conditions.

### 2.15. Soft Agar Colony Formation Assay

Soft agar assays were constructed in 6-well plates. The base layer of each well consisted of 2 mL with final concentrations of 1x completed culture media (DMEM (DU145 and HeLa), K-SFM (RWPE-1)) and 0.5% low melting point agarose (Invitrogen, 16520050). After chilling the base layers at 4°C until solid, a 1 mL growth agar layer was poured, consisting of 200 cells suspended in corresponding completed culture media and 0.2% low melting point agarose. After chilling the growth agar layer, another 1 mL corresponding completed culture media without agarose was added on top of the growth agar layer. Cells were allowed to grow at 37°C for two weeks, and the colonies were observed by a microscope.

### 2.16. Statistical Analysis

All statistical tests were done using Microsoft Excel. The statistical analysis of data was indicated in each figure. For comparison between two individual groups, standard Student's *t*-test was used. If not specifically noted, the sample group was compared to its corresponding control group. Parallel experiments were also indicated in each figure. Data were presented as means ± SD (indicated within each figure). *P* values less than 0.05 (^∗^ *P* < 0.05) were considered statistically significant.

### 2.17. mRNA Sequencing Data

The accession number for the RNA sequencing data reported in this paper is GEO: GSE112007.

## 3. Results and Discussion

### 3.1. The Global mRNA Sequencing Indicates Alterations in the Expression of Numerous Key Signaling Proteins in a Cancer Cell upon SOD1 Inhibition

**Figure 1:**
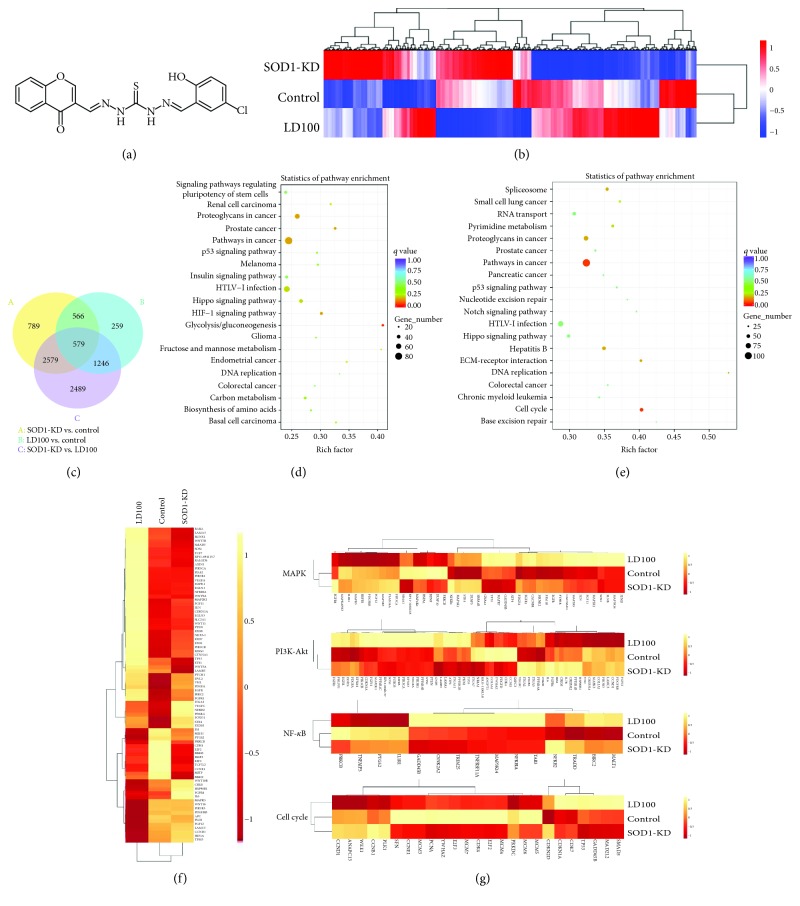
The specific SOD1 inhibition alters gene expression of numerous key signaling proteins in HeLa cells. (a) LD100, a specific SOD1 inhibitor. (b) The cluster analysis of DEGs. Based on normalized log_10_(FPKM+1), the heat map is drawn using red and blue for high-expressed genes and low-expressed genes, respectively. (c) A Venn diagram showing the overlaps between the differentially expressed genes (DEGs) in control, LD100-treated, and SOD1 knockdown HeLa cells. (d, e) Enriched KEGG pathway scatterplots of DEGs in the SOD1 inhibition group (d) and SOD1 knockdown group (e). The scatterplot shows the top 20 signaling pathways with a high level of statistical significance. (f) The cluster analysis of DEGs in signaling pathways of a cancer cell. Based on normalized log_10_(FPKM+1), the heat map is drawn using yellow and red for high-expressed genes and low-expressed genes, respectively. (g) The cluster analysis of DEGs in MAPK, PI3K-Akt, NF-*κ*B, and cell cycle signaling pathways. Based on normalized log_10_(FPKM+1), these heat maps are drawn using yellow and red for high-expressed genes and low-expressed genes, respectively.

To thoroughly understand the effects of the specific SOD1 inhibition with the inhibitor LD100 (Figure 1(a)) on the expression of the global proteome in the cancer cell, we first performed the global mRNA sequencing on the HeLa cells treated with and without 50 *μ*M LD100, and SOD1 activity was validated to be reduced by approximate 85% by this inhibitor [44]. The SOD1 knockdown group was set for comparison, and both the expression and protein levels of SOD1 were validated to be reduced by >80% in the SOD1 siRNA-treated HeLa cells after interference for 24 h relative to control (Figures S1(a) and S1(b)). The assessment of mRNA integrity prior to sequencing showed that the RNA integrity numbers of all samples were over 9 (Figure S1(c)). The *R* ^2^ values of Pearson correlations between parallel samples were over 0.98 (Figure S1(d)), a demonstration of high reproducibility of data in three parallel samples. Because all of the parallel samples met the sequencing requirements, they were successfully sequenced (Figure S1(e)), and the high-throughput sequencing data were reliable.

After mapping the reads of all mRNA sequencing samples in HeLa cells to the reference genome (Table S1), the total of 4513 differentially expressed genes (DEGs) was identified in the knockdown group and the total of 2650 DEGs was identified in the SOD1 inhibition group compared to the control group (Figure 1(b) and Figure S1(f)). The DEGs in the SOD1 knockdown group were much more than those in the SOD1 inhibition group, as SOD1 had been proposed to act as a nuclear transcription factor [26] and its specific inhibition altered only the intracellular ROS levels [44] and might not alter its activity as a nuclear transcription factor. However, 1145 overlapped DEGs in these two groups indicated that the expression of these genes was mainly regulated by the alterations of ROS levels resulted from the reduction of SOD1 activity (Figure 1(c)). Indeed, the expression of a large number of genes was found to be regulated in response to thiol peroxidase null-mediated changes of H_2_O_2_ levels in *Saccharomyces cerevisiae* cells [58]. Detailed information of the Gene Ontology (GO) classification and the Kyoto Encyclopedia of Genes and Genomes (KEGG) pathway enrichment in the SOD1 inhibition and knockdown groups was listed in Tables S2 and S3. Functions of the genes regulated in the inhibition and knockdown groups were abundant, mainly including various signaling pathways, in particular, those in onset and development of various kinds of cancer and glycolysis/gluconeogenesis (Figures 1(d) and 1(e)). In fact, SOD1 knockout was also reported to alter murine hepatic glycolysis, gluconeogenesis, and lipogenesis [59], and the changes in SOD1 expression or activity were correlated with the malignant transformation of cells [60].

Deep examination of the DEGs in the SOD1 inhibition group was performed to understand the roles of SOD1 in negative or positive control of cancer cell growth. Analysis of the KEGG pathway enrichment indicated that the specific inhibition of SOD1 significantly modified the expression of over 80 key genes in multiple signaling pathways, in particular, MAPK, PI3K-Akt, NF-*κ*B, and p53 in the cancer cell (Figures 1(f) and 1(g) and Table S3). In addition, the expression of many hallmark proteins in both cell cycle arrest and apoptosis pathways was also found to be regulated by LD100 (Figures 1(f) and 1(g) and Table S3). Because the exposure to LD100 was observed to be capable of elevating O_2_^•−^ and decreasing H_2_O_2_ levels within the cell [44], the alteration in intracellular ROS levels could regulate multiple signaling pathways that involve antiproliferation, cycle arrest, and apoptosis of cancer cells.

43 DEGs in MAPK signaling pathways were found in the SOD1-inactivating HeLa cell (Figure 1(g)). Among these genes, the most notable was PTPRR (protein tyrosine phosphatase, receptor type R) that regulates the dephosphorylation of ERK (extracellular signal-regulated kinase, one of the MAPKs). The expression of PTPRR was upregulated to 1.77 times, but the expression of PRKCB (protein kinase C beta) was downregulated to 0.21 times of that of the control (Figure S1(g)). Moreover, PRKCB was activated by the second messenger DAG (diacylglycerol) and involved ERK phosphorylation. The production of DAG was reduced because the expression of phospholipases including PLCXD3 (phosphatidylinositol-specific phospholipase C, X domain containing 3) and PLCD4 (phospholipase C-delta-4) that catalyze the formation of DAG was considerably downregulated (Figure S1(g)). Thus, the activation of PRKCB became less in the SOD1 inhibition group. Similarly, the upregulation of PTPRR and downregulation of PRKCB, PLCXD3, and PLCD4 were also observed in the SOD1 knockdown HeLa cells (Figure S1(g)). These results implied that the inactivation of ERK pathways was caused by the upregulated PTP and downregulated PKC in cells whose the H_2_O_2_ level was significantly decreased by the specific SOD1, which was not parallel to the previous conclusion that ERK pathways were regulated by the H_2_O_2_-mediated oxidative inactivation of PTPs [3, 6–9].

Similarly, the expression of 63 genes in PI3K-Akt pathways was also changed in the cell of SOD1 inhibition (Figure 1(g)). Many members in the PP2A (protein phosphatase 2A) family, which involves negative control of cell growth and division by inhibiting Akt phosphorylation, were notably upregulated compared to the control (Figure S1(g)). Moreover, the other two Akt's suppressors are as follows: PHLPP1 (pH domain-containing family E member 1) which promotes apoptosis by dephosphorylating and inactivating Akt was elevated to 1.3-fold and a PI-3,4,5-P3 phosphatase, a member of the PTEN family that negatively regulates the PI3K-Akt pathway, was upregulated to 1.3-fold of that in the control (Figure S1(g)). SOD1 knockdown was also found to suppress PI3K-Akt pathways through upregulation of several members in the PP2A family and PHLPP1 (Figure S1(g)). These data showed that the expression of many members in PP families was significantly upregulated by the treatment with LD100 or SOD1 knockdown. In addition, the suppression of PI3K-Akt pathways through SOD1 inhibition led to both downregulated mRNA levels of HIF-1*α* and 15 DEGs in NF-*κ*B pathways (Figure 1(g) and Figure S1(g)), indicating the repression of NF-*κ*B pathways.

The SOD1 inhibition also interfered with the cell cycle, as indicated by 25 DEGs observed in the LD100-treated cell (Figure 1(g)). A key step in DNA replication is assembly of the heterohexameric minichromosome maintenance (MCM2-7) helicase complex at replication origins. The reduced expression of MCM3-7 (Figure S1(h)) demonstrated that the DNA replication was attenuated and the proportion of cells in the S phase was decreased upon SOD1 inhibition. Furthermore, both reduced mRNA levels of cell cycle-associated proteins (CDK4, CCND1, CCNE1, and CCNB1) and elevated mRNA levels of CDK inhibitors (CDKN1A and CDKN2D) (Figure S1(h)) suggested that the LD100-treated cell was arrested in the G1 phase. In addition, SOD1 knockdown also confirmed the reduced expression of MCM3-7, CDK4, and CCNE1 and elevated expression of CDKN1A and CDKN2D (Figure S1(h)).

Furthermore, the expression of proteins to promote apoptosis was examined in the LD100-treated cell. First, the specific SOD1 inhibition was observed to significantly upregulate both the cancer suppressor protein p53 and downstream many key proteins in p53 signaling pathways (Figure 1(g)). Then, FOXO transcription factors were shown to have increased mRNA levels due to the SOD1 inhibition (FOXO1 and FOXO3) and knockdown (FOXO1)-mediated repression of ERK and PI3K-Akt pathways (Figure S1(h)). Obviously, the elevated expression of these proteins facilitated apoptosis of the LD100-treated cell.

Combining the above-described results suggested that the typical signaling pathways ERK, PI3K-Akt, and NF-*κ*B to support cancer cell growth were repressed and the signaling pathways to promote cell cycle arrest and apoptosis including p53 were induced by the specific SOD1 inhibition-mediated O_2_^•−^ elevation and H_2_O_2_ reduction within the cancer cells, supported by SOD1 knockdown. These regulated pathways constituted an ROS signaling network that might determine the fate of cells.

### 3.2. The ROS Signaling Pathways Are Selectively Regulated Only in Cancer Cells upon SOD1 Inhibition

To examine if the ROS signaling pathways are selectively regulated only in cancer cells upon SOD1 inhibition, intracellular LD100 and ROS levels and SOD1 activity were first evaluated. A significant difference was not found in the uptake of LD100 between DU145 and RWPE-1 (Figure S2(a)). SOD1 activity in DU145 cells was observed to be about 2.7-fold that of RWPE-1 cells, but a significant difference in O_2_^•−^ levels was not found between LD100-untreated DU145 and RWPE-1 cells. The treatment with LD100 led to reduced SOD1 activity, in DU145 (~70%) and RWPE-1 (~65%) cells (Figure S2(b)), and increased O_2_^•−^ levels in two types of cells (Figure S2(c)). However, the H_2_O_2_ content in DU145 cells was ~4-fold that in RWPE-1 cells. The dramatically decreased H_2_O_2_ content (<0.35-fold) was observed in DU145 cells following inhibition of SOD1 with LD100 (Figure S2(d)).

**Figure 2:**
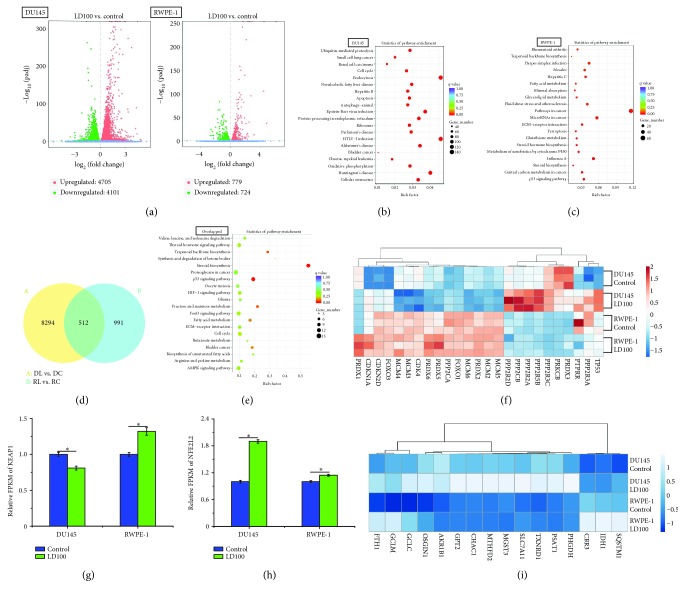
Many key genes essential for proliferation of cancer cells are selectively regulated upon SOD1 inhibition. (a) Differential mRNA expression in LD100-treated DU145 and RWPE-1 cells displayed as a volcano plot of log_2_(fold change) vs. the -log_10_(padj) for each gene. Genes considered significant below padj < 0.05 are highlighted in red and green for upregulation and downregulation, respectively. (b, c) Enriched KEGG pathway scatterplots of DEGs in LD100-treated DU145 cells (b) and RWPE-1 cells (c). The scatterplot shows the top 20 signaling pathways with high levels of statistical significance. (d) A Venn diagram showing the overlaps between the differentially expressed genes (DEGs) in LD100-treated DU145 and RWPE-1 cells. (e) An enriched KEGG pathway scatterplot of overlapped 512 DEGs. The scatterplot shows the top 20 signaling pathways with high levels of statistical significance. (f) Heat map of key DEGs in LD100-treated DU145 and RWPE-1 cells. Based on normalized log_10_(FPKM+1), the heat map is drawn using red and blue for high-expressed genes and low-expressed genes, respectively. (g) Relative FPKM data of KEAP1 in LD100-treated DU145 and RWPE-1 cells. (h) Relative FPKM data of NFE2L2 in LD100-treated DU145 and RWPE-1 cells. (i) A heat map of upregulated antioxidant proteins in representative Nrf2 pathways of LD100-treated DU145 cells. Based on normalized log_10_(FPKM+1), the heat map is drawn using white and blue for high-expressed genes and low-expressed genes, respectively. Data (g, h) are mean of triplicate samples ± SD (^∗^ *P* < 0.05; unpaired Student's *t*-test), and all error bars are SD.

Then, we determined the transcriptional response of human normal (RWPE-1) and cancer (DU145) prostate cells to the treatment with and without 50 *μ*M LD100 by the global mRNA sequencing, because SOD1 inhibition as a therapeutic method of prostate cancer has entered phase 2 clinical trials [61, 62]. Global mRNA sequencing on the DU145 and RPWE-1 cells treated with and without 50 *μ*M LD100 was performed. The RNA integrity numbers and *R* ^2^ values of Pearson correlations between parallel samples indicated that all samples met the sequencing requirements (Figures S3(a) and S3(b)) and were successfully sequenced, and the high-quality, high-throughput, and high-reproducible sequencing data were obtained (Figure S3(c)). After mapping the reads of all mRNA sequencing samples in DU145 and RWPE-1 cells to the reference genome (Table S4), 8806 DEGs were identified in the LD100-treated DU145 cells compared to the control, but only 1503 DEGs were identified in the LD100-treated RWPE-1 cells compared to the control (Figure 2(a)), indicating that the specific SOD1 inhibition could alter the expression of much more genes and affect much more biological processes in cancer cells than those in normal cells. Furthermore, the detailed information of the GO classification and KEGG pathway enrichment in the SOD1 inhibition groups of DU145 and RWPE-1 cells was listed in Tables S5 and S6. Similar to HeLa cells, the essential functions of DEGs found in SOD1-inactivated DU145 cells mainly included the signaling pathways that controlled cancer onset and development and cell apoptosis and cycle (Figure 2(b)). However, in SOD1-inactivated RWPE-1 cells, only a few of DEGs had been enriched to cancer-linked signaling pathways, and there was no significant enrichment of DEGs that could be correlated with cell apoptosis and cycle (Figure 2(c)). In addition, 512 overlapped DEGs in SOD1-inactivated DU145 and RWPE-1 cells indicated that the expression of a body of genes was regulated in both cancer and normal cells upon the specific inhibition of SOD1 (Figure 2(d)). However, the KEGG pathway enrichment analysis showed that these genes whose expression was simultaneously changed did not involve signaling pathways and biochemical processes to control the fate of cells with an exception that only a few of genes among them could be associated with p53 signaling pathways and steroid biosynthesis (Figure 2(e)).

To further understand the selectively triggered cycle arrest and apoptosis of SOD1-inactivated cancer cells, deep analysis of the DEGs was carried out for LD100-treated DU145 and RWPE-1 cells. 274 DEGs in signaling pathways, in particular, those in MAPK, PI3K-Akt, and NF-*κ*B signaling pathways, were found much more in DU145 cells than 76 DEGs in RWPE-1 cells under the SOD1-inactivated conditions (Figures S4 and S5), indicating that the signaling pathways essential for cancer cell growth were much more sensitive to the specific SOD1 inhibition than those for normal cells. Similar to the results observed in the HeLa cells, SOD1 inhibition repressed ERK pathways in DU145 cells through both upregulation of PTPRR and downregulation of PRKCB (Figure 2(f)), whereas PTPRR was slightly downregulated and PRKCB was expressed at a very low level in RWPE-1 cells exposed to LD100 (Figure 2(f)). These were a demonstration that the ERK pathways were selectively repressed via both upregulation of PTPRR and downregulation of PRKCB only in cancer cells. For PI3K-Akt pathways, the expression of seven PP2A family members was upregulated in DU145 cells, but any member of the PP2A family was not significantly altered in RWPE-1 cells (Figure 2(f)). Because the suppression of PI3K-Akt pathways could block NF-*κ*B pathways, NF-*κ*B pathways were also inhibited in DU145 cells, as indicated by 46 DEGs in NF-*κ*B pathways (Figure 4(d)). Therefore, the signaling pathways crucial for malignant tumorigenesis were selectively repressed in cancer cells upon the specific SOD1 inhibition.

The DEGs responsible for the cell cycle were found to be 82 in DU145 cells, but only 18 in RWPE-1 cells, when exposed to LD100 (Figure S6(a)). For example, the reduced expression of MCM2-6 proteins indicated that the exposure to LD100 halted DU145 cells in the S phase by inhibiting DNA replication, whereas DNA replication was not affected in LD100-treated RWPE-1 cells because of unchanged expression of MCMs (Figure 2(f)). Furthermore, although LD100 was not found to have impact on the expression of CDK4 and CDK inhibitors (CDKN1A and CDKN2D) in the RWPE-1 cells, the downregulation of CDK4, as well as the upregulation of both CDK inhibitors and the cell cycle arrest gene Gadd45, suggested that DU145 was halted in the G1 phase of the cell cycle (Figure 2(f) and Figure S7). Therefore, SOD1 inhibition could result in cycle arrest of cancer cells, not normal cells.

The specific SOD1 inhibition in DU145 cells upregulated the expression of p53 (Figure 2(f)) and activated much more genes in p53 signaling pathways, in contrast to that in RWPE-1 cells (Figure S6(b)). These activated genes involved cell cycle arrest, acceleration of mitochondria-dependent apoptosis, repair and prevention of DNA damage, repression of IGF-1/mTOR pathways, and activation of the p53 negative feedback (Figure S7). In fact, 87 DEGs were found to involve apoptosis in LD100-treated DU145 cells, not in RWPE-1 cells (Figure S6(c)). Moreover, the upregulation of FOXO1 and FOXO3, which can be elevated by the repression of ERK and PI3K-Akt pathways, was also observed only in the SOD1-inactivated cells (Figure 2(f)). The induction of p53 signaling pathways and elevated expression of these genes suggested that SOD1 inhibition could selectively promote apoptosis of cancer cells in a mitochondria-dependent manner, supported by the induction of the genes DR5, Noxa, PUMA, and Siah (Figure S7).

In addition, Nrf2 (also named NFE2L2) regulates the expression of antioxidant proteins to protect organisms against oxidative damage, but the binding of KEAP1 inactivates Nrf2. The Nrf2 activation is controlled by FOXOs and p53 via regulation of CDKN1A expression [63]. In LD100 treated-DU145 cells, both the activation of p53 pathways and upregulation of FOXOs increased Nrf2 mRNA levels via elevating CDKN1A expression, and the downregulation of KEAP1 led to increased activation of Nrf2 (Figures 2(f)–2(h)), resulting in the increased expression of 16 antioxidant proteins in representative Nrf2 pathways (Figure 2(i)). However, the unchanged FOXOs and upregulated KEAP1 indicated the inactivation of Nrf2 antioxidant pathways in RWPE-1 cells under the tested conditions (Figures 2(f) and 2(g)). Thus, the specific SOD1 inhibition stimulated Nrf2 antioxidant pathways through changing relative ROS levels.

In summary, the above-described results suggested that specific SOD1 inhibition repressed the ROS signaling pathways crucial for malignant tumorigenesis and stimulated the signaling pathways to promote both cell cycle arrest and apoptosis through regulation of the expression of numerous genes in cancer cells, whereas all of these signaling pathways were not found to be significantly changed in the SOD1-inactivated normal cells.

### 3.3. SOD1 Inhibition Represses the Typical ROS Signaling Pathways in Cancer Cells

**Figure 3:**
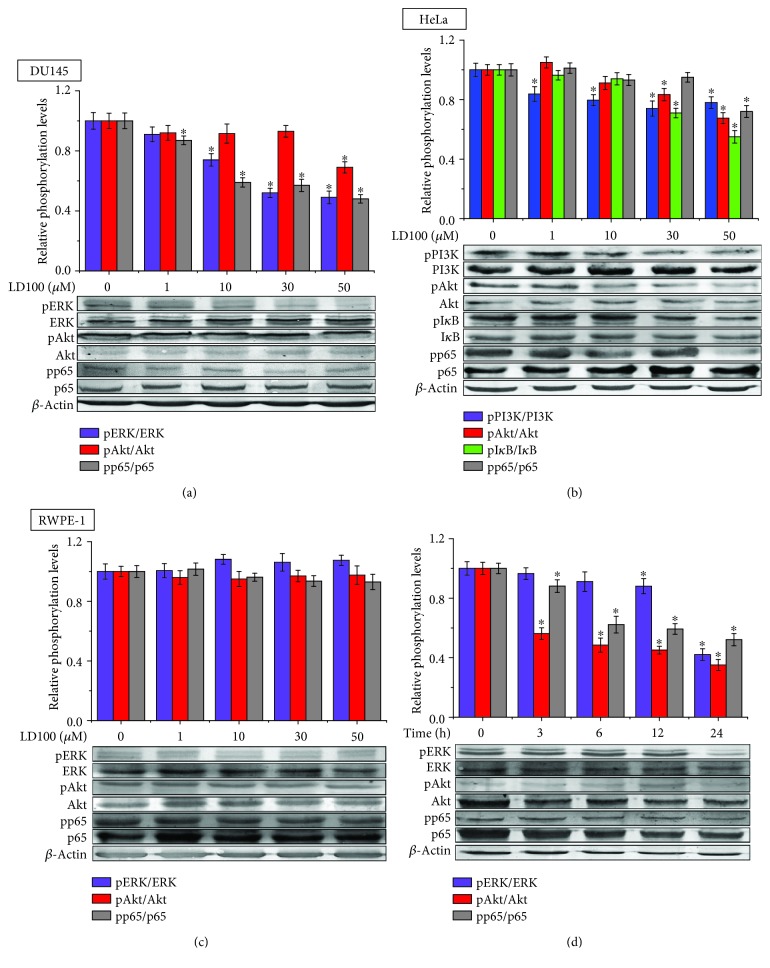
The specific SOD1 inhibition selectively represses ERK, PI3K, and NF-*κ*B signaling pathways in cancer cells. (a–c) The phosphorylation of the key signaling proteins is evaluated by western blotting analysis. DU145, HeLa, and RWPE-1 cells are incubated, respectively, with varied concentrations of LD100 for 24 h. (d) The time dependence of the signaling protein phosphorylation in 50 *μ*M LD100-treated HeLa cells is evaluated by western blotting analysis. The relative intensity of protein bands (means ± SD) in the western blotting is quantified by using the ImageJ software and normalized through the negative control, respectively. Representative results from three independent experiments are shown. Data are mean of triplicate samples ± SD (^∗^ *P* < 0.05; unpaired Student's *t*-test), and all error bars are SD.

To confirm that the specific SOD1 inhibition represses the typical ROS signaling pathways ERK, PI3K, and NF-*κ*B in cancer cells, we tested dependences of the phosphorylation of the key proteins in these pathways on varied concentrations and incubation times of LD100, respectively, added into HeLa, prostate normal, and cancer cells. First, western blotting tests showed that the phosphorylation of the signaling proteins ERK, PI3K, Akt, I*κ*B, and p65 (a subunit of NF-*κ*B) was significantly reduced, respectively, with the concentration of 0-50 *μ*M LD100 in these two types of cancer cells, e.g., 50% of reduction for pERK [44], 75% for pPI3K, 65% for pAkt, 55% for pI*κ*B, and 70% for pp65 at 50 *μ*M LD100 in HeLa cells relative to controls after incubation for 24 h, although levels of these proteins remained unchanged in this concentration range of LD100 (Figures 3(a) and 3(b)). However, the phosphorylation of the signaling proteins ERK, Akt, and p65 was not modified with the concentration of LD100 in the prostate normal cell RWPE-1 under the tested conditions (Figure 3(c)). Obviously, these results indicated that the ROS signaling pathways were repressed in a SOD1 inhibitor concentration-dependent fashion only in cancer cells. Then, the time dependence of the phosphorylation of the signaling proteins was tested in HeLa cells incubated with 50 *μ*M LD100 under the tested conditions. The data showed that levels of the phosphorylated signaling proteins were progressively decreased in this type of cancer cells with prolonging incubation (0-24 h) with LD100 (Figure 3(d)), indicating that the repression of the ROS signaling pathways was increasingly enhanced with prolonging time of the specific SOD1 inhibition in cancer cells.

Our previous tests revealed that O_2_^•−^ and H_2_O_2_ levels were elevated and decreased, respectively, in cancer cells with increasing concentrations of LD100 and prolonging time of incubation with LD100 [44]. Here, the obtained results indicated that the phosphorylation of the key proteins in the signaling pathways ERK, PI3K, and NF-*κ*B was reduced with the relative alterations in ROS levels only in the cancer cells. Hence, we concluded that the ROS signaling pathways were increasingly repressed with the elevation in O_2_^•−^ and reduction in H_2_O_2_ levels in cancer cells, but not in the normal cell.

### 3.4. SOD1 Inhibition Impacts Crosstalk among the Typical ROS Signaling Pathways in Cancer Cells

The repression of the typical ROS signaling pathways by the specific SOD1 inhibition inspires us to hypothesize that LD100 might impact their crosstalk in cancer cells. To support this hypothesis, we examined LD100 regulation of the signaling pathways in cancer cells when they were inhibited, respectively or simultaneously, with the commercial inhibitors. Here, the used inhibitors U0126, LY294002, and BAY117082 targeted, respectively, ERK, PI3K, and NF-*κ*B pathways. First, we tested the impact of the SOD1 inactivation on the crosstalk between ERK and PI3K pathways by exposing HeLa cells to 50 *μ*M LD100, 10 *μ*M U0126, and LY294002 in an orthogonal fashion for 24 h. The respective and simultaneous addition of LD100 and U0126 both attenuated ERK phosphorylation, although the level of ERK proteins remained unchanged in these tests. However, the presence of LY294002 elevated ERK phosphorylation to 1.45-fold of the control, and readdition of LD100 restored the ERK phosphorylation to the reduced level (Figure S8(a)). These results indicated that the ERK pathway could compensate the lost activity of the PI3K pathway due to the inhibition stemmed from LY294002 and the specific inactivation of SOD1 did not facilitate the crosstalk between ERK and PI3K pathways. Under the tested conditions, the respective and simultaneous presence of LD100 and LY294002 both also attenuated the phosphorylation of PI3K and Akt (Figures S8(b) and S8(c)). However, U0126 notably elevated the phosphorylation of both, respectively, to 1.25- and 1.3-fold of the control, but LD100 led to this elevated phosphorylation to restore the reduced level. These observations were a demonstration that the specific SOD1 inhibition could interfere with the crosstalk between ERK and PI3K-Akt pathways by blocking the feedback regulation between ERK and PI3K or ERK and Akt.

Furthermore, the impact of the SOD1 inhibition on the crosstalk among the pathways ERK, PI3K, and NF-*κ*B was examined by exposure of cells to 50 *μ*M LD100, 10 *μ*M U0126, LY294002, and BAY117082 in an orthogonal fashion for 24 h. The examination showed that (1) the phosphorylation of NF-*κ*B subunit p65 was reduced by the respective and simultaneous exposure to LD100, LY294002, and BAY117082, not by exposure to U0126 (Figure S8(d)), (2) NF-*κ*B positioned at the downstream of PI3K-Akt, and (3) the pathway ERK was parallel to the PI3K-Akt and NF-*κ*B signaling cascade. Taken together with the above-described results, the specific SOD1 inhibition-mediated alterations of ROS levels blocked the feedback regulation mediated by the specific pathway inhibitors among ERK, PI3K, and NF-*κ*B through simultaneously repressing two pathways, resulting in the interruption of furthermore transmission of the signals within cancer cells.

### 3.5. SOD1 Inhibition Leads to Cancer Cell Cycle Arrest

**Figure 4:**
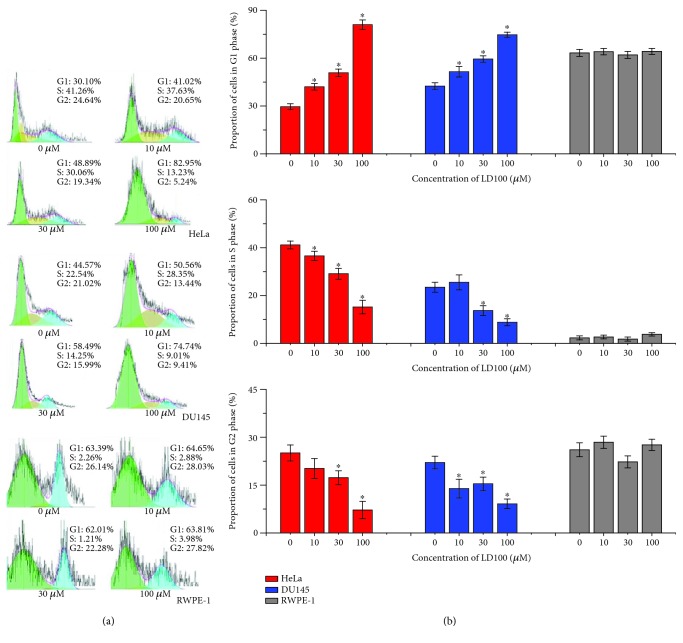
The specific SOD1 inhibition selectively arrests the cell cycle of cancer cells. HeLa, DU145, and RWPE-1 cells are incubated, respectively, with varied concentrations of LD100 for 24 h prior to analysis of cell distributions in different cycle phases by flow cytometry using propidium iodide staining. To obtain more significant results, 100 *μ*M LD100 was used here. Representative results from three independent experiments are shown in (a). Data are mean of triplicate samples ± SD (^∗^ *P* < 0.05; unpaired Student's *t*-test), and all error bars are SD.

**Figure 5 fig5:**
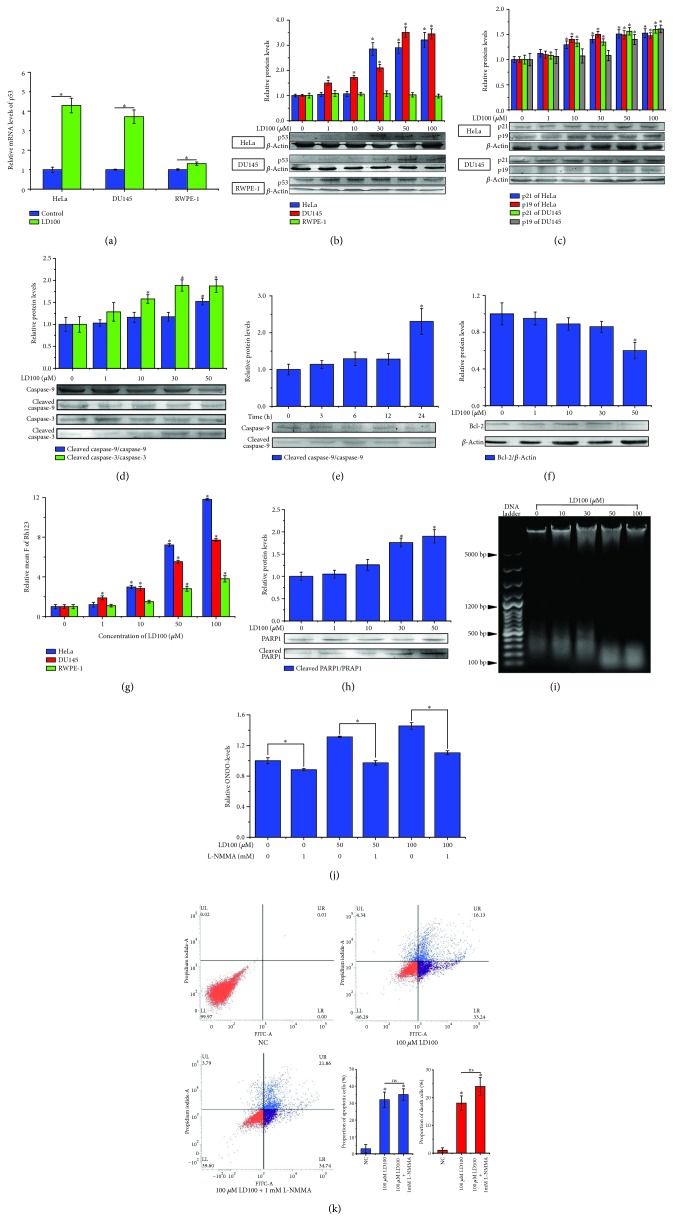
The specific SOD1 inhibition activates mitochondria-dependent apoptosis of cancer cells. (a, b) RT-qPCR (a) and western blotting (b) examination of p53 mRNA levels and protein content in 50 (RT-qPCR) or 0-100 *μ*M (western blotting) LD100-treated HeLa, DU145, and RWPE-1 cells. (c) The protein levels of p21 and p19 in HeLa and DU145 cells are evaluated by western blotting analysis, after the cells are incubated with varied concentrations of LD100 for 24 h. *β*-Actin is used as an internal control (b, c). (d) The protein levels of proto- and cleaved caspase-9 and -3 in HeLa cells are evaluated by western blotting analysis, after HeLa cells are incubated with varied concentrations of LD100 for 24 h. (e) The time dependence of the protein levels of proto- and cleaved caspase-9 is evaluated by western blotting analysis in 50 *μ*M LD100-treated HeLa cells. (f, h) HeLa cells are incubated with varied concentrations of LD100 for 24 h prior to western blotting evaluation of Bcl-2 (f) and cleaved PARP1 (h) protein levels. (g) Rh123 fluorescence that is positively correlated with mitochondrial membrane potential is measured by flow cytometry in HeLa, DU145, and RWPE-1 cells after incubated with varied concentrations of LD100 for 24 h. (i) DNA fragmentation in HeLa cells is assayed using agarose gel electrophoresis after incubated with varied concentrations of LD100 for 24 h. (j) The peroxynitrite content in HeLa cells is presented by DHR123 fluorescence measured with flow cytometry after incubation for 24 h, respectively, with LD100 (0, 50, and 100 *μ*M) and L-NMMA (0, 1 mM), a cell membrane-permeable competitive NOS inhibitor. (k) After incubation for 24 h, respectively, with LD100 (0, 100 *μ*M) and L-NMMA (1 mM L-NMMA), flow cytometric analysis of HeLa cell apoptosis is carried out with Annexin V and propidium iodide staining. To obtain more significant results, 100 *μ*M LD100 was used here. The relative intensity of protein bands (means ± SD) in the western blotting is quantified by using the ImageJ software and normalized through the negative control, respectively (b–g). Representative results from three independent experiments are shown (b–g and k). Data are mean of triplicate samples ± SD (ns: not statistically significant; ^∗^ *P* < 0.05; unpaired Student's *t*-test), and all error bars are SD.

The activation of ROS signaling pathways including ERK promoted growth of cancer cells through controlling the expression of cell cycle proteins. The global mRNA sequencing data indicated that the expression of many protein families that sustained a normal cell cycle was significantly downregulated in the LD100-treated cancer cells (Figures 1(g) and 2(f) and Figures S1(h) and S6(a)), suggesting that the specific SOD1 inhibition led to cancer cell cycle arrest. In support of this anticipation, we observed the impact of increasing LD100 concentrations on cycles of three types of cells. The cytometric data showed that the proportion of HeLa and DU145 cells was increased in the G1 phase, but as a general trend decreased in S and G2 phases with increasing concentrations of LD100 after 24 h incubation (Figure 4), which was well consistent with the anticipation stemmed from the global mRNA sequencing (Figures 1(g) and 2(f) and Figures S1(h) and S6(a)). In fact, the elevated levels of three cycle inhibitors p53, p21 (CDKN1A), and p19 (CDKN2A) confirmed the arrest of these two types of cancer cells in the G1 phase (Figures 5(a), 5(b), and 5(c)). Moreover, comparison of the data indicated that LD100 retarded less growth of DU145 than that of HeLa cells likely owing to their different characteristics of growth. However, the cell cycle was not found to be compromised for RWPE-1 cells in the tested concentration range of LD100 (Figure 4). In fact, the global mRNA sequencing data demonstrated that the expression of many cell cycle proteins was not significantly modified in RWPE-1 cells compared to that both in HeLa and in DU145 cells upon the SOD1 inactivation (Figures 1(g) and 2(f) and Figures S1(a) and S6(a)). Overall, these results revealed that the specific SOD1 inhibition promoted cancer cell cycle arrest, resulting in the retardation of cancer cell growth, but do not impact normal cell growth. The reasons were that cancer cells needed high levels of H_2_O_2_ to support their growth [10–14], whereas the SOD1 inhibition efficiently decreased intracellular levels of H_2_O_2_, leading to the interruption of ROS homeostasis in cancer cells [44].

### 3.6. SOD1 Inhibition Activates Apoptosis of Cancer Cells

The cell cycle arrest and repression of the ROS signaling pathways indicated that the specific SOD1 inhibition could activate the signaling pathways to promote cancer cell apoptosis. To prove this anticipation, we tested alterations in levels of the representative markers in apoptosis of the cancer cells treated with varied concentrations of LD100. First, the expression of cancer's suppressor p53 was upregulated to >3.5-fold of the control in two types of cancer cells, but remained almost unchanged in the prostate normal cells, as indicated by its mRNA data (Figure 5(a)). Moreover, as a consequence of the prevention of p53 degradation by the LD100 treatment-mediated elevation in the expression of p19, the increase in the protein content of p53 was more pronounced in the cancer cells than in the normal cells relative to the control, whereas the increased p53 content resulted in elevation in the cycle inhibitor p21 level, as indicated by western blotting tests (Figures 5(b) and 5(c)). These results demonstrated that the specific SOD1 inhibition could suppress the growth of cancer cells via the upregulation of p53 expression. Then, an important pair of members in the apoptosis protein family of caspase, caspases 3 and 9 that target downstream proteins to induce apoptosis, was examined in the HeLa cells. This pair of proteins essential for the initiation and development of apoptosis occurs in the proto- and proteolytically activated forms in a cell. The activated forms of caspases 3 and 9 can act on their substrates. The tests showed that the intracellular content of activated caspase 9 was increased and its prototype was reduced with rising LD100 concentrations after incubation for 24 h (Figure 5(d)) or prolonging time of incubation with 50 *μ*M LD100 in the cancer cell (Figure 5(e)). The activation of caspase 9 was very pronounced at the high LD100 concentrations, e.g., 50 *μ*M LD100 led to growth of the activated caspase 9 to 2.3-fold of the control after incubation for 24 h (Figure 5(e)). Similarly, the activated caspase 3 form was also observed to rise with the LD100 concentration and was upregulated to 1.85-fold of the control after incubation with 50 *μ*M LD100 for 24 h in the cancer cell, whereas the content of caspase 3 remained unchanged (Figure 5(d)). These results indicated that LD100 upregulated the activation of these two kinds of proapoptosis proteins in a concentration-dependent fashion in the cancer cell and the specific SOD1 inhibition should induce apoptosis of cancer cells through activating the mitochondria-dependent apoptosis pathway.

The level of the antiapoptosis protein Bcl-2, one member of the Bcl-X family, was analyzed in the HeLa cells under the tested conditions. Bcl-2 is essential for the maintenance of mitochondrial membrane potential by localizing at the mitochondrial outer membrane. The data showed that the intracellular Bcl-2 level was decreased in an LD100 concentration-dependent manner. In particular, Bcl-2 was pronouncedly downregulated after LD100 reaches 50 *μ*M (Figure 5(f)). These were not only a demonstration that the specific SOD1 inhibition attenuated the expression of the antiapoptosis protein by the repression of the pathway NF-*κ*B, but also an implication that the mitochondrial membranes were interrupted by the exposure to LD100.

To examine whether the mitochondrial membrane potential is maintained or not, we tested the integrity of mitochondrial membranes in HeLa, prostate cancer, and normal cells incubated with varied concentrations of LD100 for 24 h, because the potential differential between the inner and outer membranes disappears in the membrane-interrupted mitochondrion. The fluorescence intensity of rhodamine 123 (Rh123) is positively correlated with the interruption of mitochondrial membranes, reflecting the increasing disappearance of the mitochondrial membrane potential. The data showed that the fluorescence of Rh123 was significantly intensified in HeLa and prostate cancer cells when LD100 was ≥10 *μ*M, whereas the fluorescence of Rh123 was slightly intensified with the concentration of LD100 in the normal cell (Figure 5(g)), indicating that the alteration in the permeability of mitochondrial membranes was LD100 concentration-dependent in cancer cells, and the integrity of mitochondrial membranes was almost not affected by LD100 in normal cells under the tested conditions. Therefore, the specific SOD1 inhibition induced apoptosis of cancer cells, not normal cells, in a mitochondria-dependent manner.

To further prove the SOD1 inhibition-induced apoptosis of cancer cells, we analyzed the cleavage of poly(ADP-ribose)polymerase 1 (PARP1) and DNA, two key markers of apoptosis, in the cancer cell treated with varied concentrations of LD100. On one hand, PARP1, which is essential for nucleic acid repair, should be increasingly cleaved by the increased activated form of caspase 3, because it is a substrate of caspase 3. Indeed, the cleaved PARP1 was observed to noticeably rise in an LD100 concentration-dependent manner in the cancer cell, e.g., 50 *μ*M LD100 led the cleaved PARP1 to 1.9-fold of the control (Figure 5(h)), which was well consistent with the upregulation of the activated caspase 3 (Figure 5(d)). However, the undamaged PARP1 was almost not altered over the concentration range of LD100 under the tested conditions (Figure 5(h)). On the other hand, we assayed the fragmentation of DNA in HeLa cells exposed to varied concentrations of LD100, because a consequence resulted from the increased activation of caspase 3 was the rise in unrepaired DNA damage. As expected, the content of intracellular ~200 bp DNAs was significantly increased with LD100 concentrations, in particular, at ≥50 *μ*M of LD100 (Figure 5(i)). The results indicated that the specific SOD1 inhibition increased the cleaved amount of PARP1 through enhancing the activation of caspase 3, thereby resulting in the intensified fragmentation of DNAs in cancer cells.

Overall, the specific SOD1 inhibition-mediated O_2_^•−^ elevation and H_2_O_2_ reduction dramatically stimulated not only the upregulation of both the cancer suppressor p53 and the activated form of the apoptosis-regulatory protein caspase 9 but also the downregulation of the antiapoptosis protein Bcl-2, thereby triggering the programmed death of cancer cells, not normal cells. This kind of apoptosis occurred via a mitochondria-dependent pathway and was always maintained and even amplified through proteolytic activation of caspase 9 and apoptosis effector caspase 3, which was supported by the cleavage-mediated inactivation of PARP1 and intensified DNA fragmentation in cancer cells.

### 3.7. Peroxynitrite Does Not Involve the SOD1 Inhibition-Mediated Apoptosis of Cancer Cells

To further support the above-mentioned conclusion that the apoptosis of cancer cells is stemmed from the regulation of the ROS signaling network, we examined roles of peroxynitrite (ONOO^−^) in apoptosis. The specific SOD1 inhibition-mediated O_2_^•−^ elevation could result in increased production of ONOO^−^ in cancer cells, because the ready reaction of O_2_^•−^ with NO-elevated production of ONOO^−^. Peroxynitrite promoted cell apoptosis through oxidatively damaging biomacromolecules. L-NMMA is a cell membrane-permeable competitive inhibitor of NO synthase and decreases the production of NO in cancer cells. If the LD100-mediated apoptosis was originated from the elevated production of peroxynitrite, the addition of L-NMMA could compromise the LD100-mediated apoptosis.

First, we tested changes in ONOO^−^ levels in HeLa cells, respectively, exposed to LD100, L-NMMA, or both after 24 h incubation by green fluorescence of dihydrorhodamine 123 (DHR, excited at 488 nm), a sensitive probe whose fluorescent intensity is positively correlated with concentrations of ONOO^−^. The cells exposed, respectively, to 50 and 100 *μ*M LD100 were observed to display more brilliant green fluorescence compared to the control under an inverted fluorescence microscope (Figure S9), demonstrating that the specific SOD1 inhibition resulted in the increased production of ONOO^−^. The addition of 1 mM L-NMMA significantly reduced intracellular content of ONOO^−^, as indicated by the weakened DHR fluorescence (Figure S9). The cytometric data under the tested conditions indicated that the addition of L-NMMA could restore the LD100-mediated high content to the low content of ONOO^−^ in the control (Figure 5(j)), providing quantitative support to the fluorescence microscopic observation.

Then, the relationship between the elevated production of peroxynitrite and apoptosis was examined for the cancer cells exposed, respectively, to LD100, L-NMMA, or both. The cells were found to arrive at about 50% apoptosis after treatment 24 h with 100 *μ*M LD100, as previously observed [44], but the addition of 1 mM L-NMMA unexpectedly elevated the apoptosis ratio of the cells to ~56% from ~50% (Figure 5(k)). Obviously, these results ruled out the possibility that the elevated apoptosis of cancer cells exposed to LD100 was stemmed from the specific SOD1 inhibition-mediated elevated production of peroxynitrite. In addition, the elevated apoptosis in the presence of both LD100 and L-NMMA could be attributed to the excessive consumption of intracellular nitric oxide, because the reaction of superoxide anion with nitric oxide and the L-NMMA inhibition of nitric oxide synthase both could reduce levels of nitrite oxide below the physiological level that was essential for normal cell growth.

### 3.8. SOD1 Inhibition Hinders the Formation of Cancer Cell Colonies

**Figure 6:**
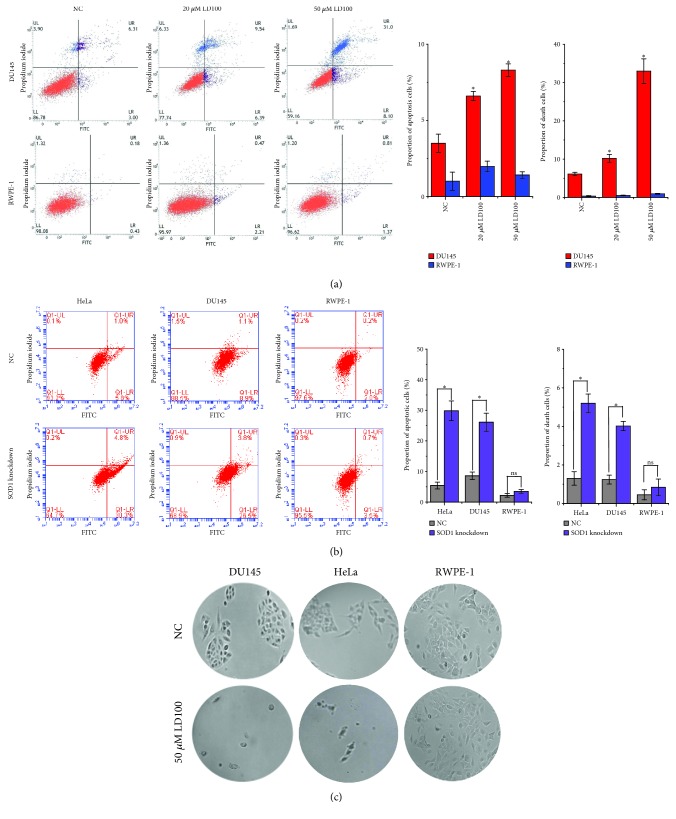
The specific SOD1 inhibition induces apoptosis and hinders the formation of cancer cell colonies. (a) After incubation for 24 h with LD100 (0, 20, and 50 *μ*M), cytometric assays of apoptosis are assessed on RWPE-1 and DU145 cells by Annexin V and propidium iodide staining. (b) After transfection with validated 35 pmol siRNA of SOD1 for 24 h, cytometric assays of apoptosis are assessed on HeLa, DU145, and RWPE-1 cells by Annexin V and propidium iodide staining. (c) Exposure to 50 *μ*M LD100 for 2 weeks hinders the formation of HeLa and DU145 cell colonies, whereas the formation of RWPE-1 cell colonies is not obviously affected. Representative results from three independent experiments are shown (a, b). Data are mean of triplicate samples ± SD (ns: not statistically significant; ^∗^ *P* < 0.05; unpaired Student's *t*-test), and all error bars are SD.

The above-observed repression and activation of ROS signaling pathways to promote growth and apoptosis of cancer cells inspired us, respectively, to assess apoptosis and colonial formation of the prostate cancer and normal cells exposed to different LD100 concentrations for 24 h. Exposure to LD100 was observed to promote apoptosis of HeLa cells [44]. As expected, the cytometric data showed that the proportion of apoptosis and death of DU145 cells rose with increasing LD100 concentrations, whereas the proportion of RWPE-1 cells was only slightly increased with LD100 concentrations (Figure 6(a)). SOD1 knockdown also selectively promoted cancer cell apoptosis (HeLa and DU145), but not normal cells (RWPE-1) (Figure 6(b)). Moreover, large colonies were not found for DU145 and HeLa cells treated with 50 *μ*M LD100 for 1-2 weeks compared to the untreated cells, but the colonial formation of the normal cells RWPE-1 was not affected by this treatment under the tested conditions (Figure 6(c)). Furthermore, significant differences were not found in the growth rate of the SOD1 inhibited and noninhibited RWPE-1 cells in 14 days (Figure S10). All these results indicated that the specific SOD1 inhibition did noticeably suppress proliferation and growth of cancer cells, not normal cells, through induction of apoptosis.

## 4. Conclusions

The specific SOD1 inhibition results in reduced H_2_O_2_ and elevated O_2_^•−^ content inside cells [44]. Although this inhibition can reduce levels of H_2_O_2_ not only in cancer cells but also in normal cells, the decreased extent of H_2_O_2_ in cancer cells is much larger than that in normal cells under the tested conditions, resulting in the disruption of ROS homeostasis in cancer cells.

The global transcriptomic data on two types of cancer and a type of normal cells indicated that the most changed expression upon this interruption of ROS homeostasis is the key genes in plenty of signaling pathways inside cancer cells, not inside normal cells, although mRNA levels of numerous genes are altered in three lines of cells (Figures 1(d), 1(f), 1(g), and 2(f)–2(i) and Figures S1(g), S1(h), and S4–S7). The modulation of gene expression leads to repression of the signaling pathways including ERK, PI3K, and NF-*κ*B and their crosstalk to promote cell growth (Figure 3 and Figure S8) and activation of the signaling pathways to cause both cell cycle arrest and apoptosis (Figures 4 [Fig fig5]–6). These results indicated that the specific inhibition of SOD1 selectively promotes apoptosis of cancer cells via regulation of the ROS signaling network, supported by the similar results obtained in SOD1 knockdown cells (Figures 1(e)–1(g) and 6(b)). The elevated peroxynitrite production resulted from the increased superoxide content does not contribute to the selective killing of cancer cells.

**Figure 7:**
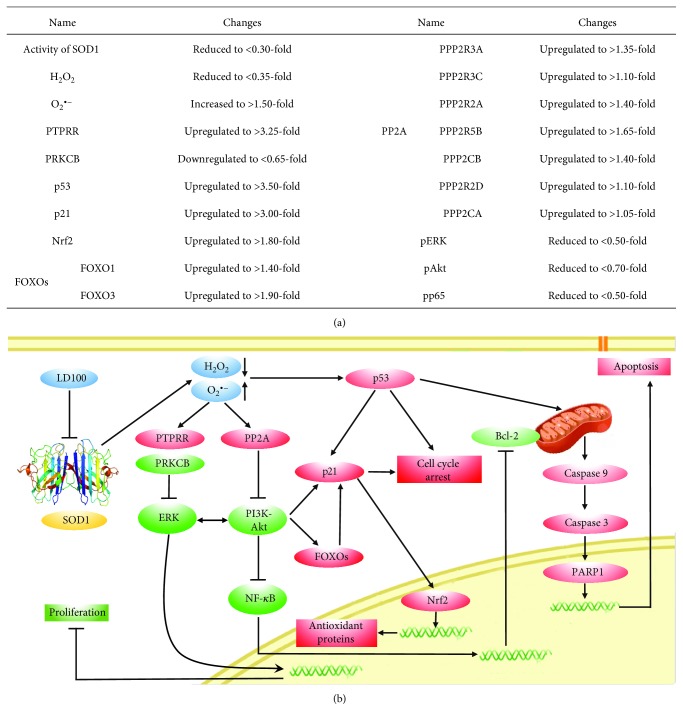
The specific inhibition reveals that SOD1 is a regulatory hub in the ROS signaling network that is consisted of the signaling pathways to support growth of cancer cells and to promote cycle arrest and apoptosis of cancer cells. (a) After treating DU145 cells with 50 *μ*M LD100 for 24 h, the main factors leading to cell cycle arrest, mitochondria-dependent apoptosis, and upregulation of antioxidant proteins are summarized. (b) SOD1 is a regulatory hub in the ROS signaling network. Here, red color represents activation or upregulation, and green color represents repression or downregulation.

The specific SOD1 inhibition-mediated reduction of H_2_O_2_ content is crucial to the selective apoptosis of cancer cells. On one hand, on the basis of the currently putative opinion, the oxidative inactivation of phosphatases including PTPs and PTENs by H_2_O_2_ at appropriately high levels triggers signaling transduction and crosstalk among signaling pathways to support uncontrollable growth of cells, resulting in tumorigenesis, angiogenesis, and metastasis of cancer cells [10–14]. The uncontrollable growth of cancer cells is attributed to the intracellular sustained high levels of H_2_O_2_ provided by upregulated expression and activity of SOD1 [48–51]. However, SOD1 in normal cells is controlled at the appropriate expression and activity levels essential for their physiological functions [15]. On the other hand, reduction in intracellular H_2_O_2_ content within cancer cells results in the interruption of the signaling pathways including ERK, PI3K, and NF-*κ*B by decreasing protein phosphorylation, but does not influence the pathways within normal cells. Although the intracellular O_2_^•−^ content is significantly elevated upon SOD1 inhibition, O_2_^•−^ cannot cause oxidative inactivation of the phosphatases [3]. In addition, the pathways of cell cycle arrest are induced as a consequence of ERK inhibition. Therefore, the specific SOD1 inhibition selectively represses the signaling pathways to sustain uncontrollable growth of cancer cells and stimulates the pathways to promote cancer cell cycle arrest (Figure 7).

Furthermore, SOD1 inhibition activates p53 signaling pathways via upregulating expression of the cell cycle inhibitors p19, p21, and p53, resulting in growth suppression and apoptosis of cancer cells in a mitochondria-dependent manner. The data showed that the activation of p53 pathways elevates the expression of Nrf2. In the past, the roles of Nrf2 in antioxidant gene expression and ROS detoxification are believed to be facets of a tumor suppressor function, although recent evidence indicates that Nrf2 promotes maintenance of cancer cell growth [63]. The apoptosis data of DU145 and RWPE-1 cells exposed to LD100 (Figure 6) provide a line of support to this tumor suppressor function of Nrf2. In addition, the alteration of intracellular ROS levels leads to both upregulations of the apoptosis-regulatory protein caspases 3 and 9 and downregulation of the antiapoptosis protein Bcl-2. However, similar phenomena are not observed in normal cells. Overall, the specific SOD1 inhibition selectively kills cancer cells through both repressions of the ROS pathways to sustain cell growth and induction of cell cycle arrest and apoptosis (Figure 7).

The results presented here inspire us to think the mechanisms that ROS activates the signaling pathways to promote cancer cell survival. There are two major schools of opinions: direct oxidation and redox relay-mediated oxidation of signaling target proteins via peroxiredoxin (Prx), aiming to explain phenomena of H_2_O_2_ signaling [64]. We propose that ROS modification of the expression of numerous signaling genes is also an explanation on ROS signaling, supported by (1) thousands of DEGs indicated not only by global mRNA sequencing data but also by the reduced expression of key genes in multiple signaling pathways including ERK, PI3K, and NF-*κ*B inside the LD100-treated and SOD1 knockdown cancer cells (Figures 1 and 2), (2) the ROS signaling network that is not modified in the LD100-treated normal cells, and (3) downregulation in the cancer cells of few PRX isoforms and upregulation of few PRX isoforms in the normal cells. This opinion is also supported by the observation that the expression of a large number of genes is regulated in response to thiol peroxidase null-mediated changes of H_2_O_2_ levels in *Saccharomyces cerevisiae* cells [58].

Many excellent findings were recently reported on the oncogenic effect of copper [65] and anticancer activity of the copper-chelating agents DDC [66] and TM. The anticancer activity and their derivatives are attributed to the formation of their copper complexes in these reports. We examined the selective cancer cell-killing effect of a specific SOD1 inhibitor (LD100) that serves as a copper-chelated agent from the modulation view of the ROS signaling network, revealing that this effect of LD100 can be attributed to the SOD1 inhibition-mediated repression of the ROS signaling pathways to promote maintenance of cancer cell growth and the activation of the ROS pathways to trigger cycle arrest and apoptosis of cancer cells, not normal cells. This examination might facilitate disclosure of action mechanisms by copper-chelated anticancer agents and design of anticancer small molecules that target SOD1 copper and interfere with the ROS signaling network.

## Data Availability

The data used to support the findings of this study are available from the corresponding author upon request.

## Supplementary Materials

Supplementary Materials Figure S1: the levels of SOD1 in SOD1 knockdown HeLa cells and key DEGs in LD100-treated HeLa cells. Figure S2: SOD1 activity and H_2_O_2_ and O_2_^•−^ levels within LD100-treated DU145 and RWPE-1 cells. Figure S3: successful construction of mRNA sequencing in LD100-treated DU145 and RWPE-1 cells. Figure S4: many key genes in representative signaling pathways are selectively regulated upon the specific SOD1 inhibition. Figure S5: the specific SOD1 inhibition selectively regulates cancer pathways in cancer cells. Figure S6: many key genes in the cell cycle and p53 and apoptosis signaling pathways are selectively regulated upon the specific SOD1 inhibition. Figure S7: the specific SOD1 inhibition selectively activates the p53 signaling pathway in cancer cells. Figure S8: specific SOD1 inhibition represses the crosstalk of ERK, PI3K, and NF-*κ*B signaling pathways in cancer cells. Figure S9: the specific SOD1 inhibition increases the intracellular peroxynitrite content. Figure S10: the specific SOD1 inhibition does not affect the growth and proliferation of normal cells. Table S1: quality evaluation of transcriptome library in HeLa cells. Table S2: GO classification of DEGs in LD100-treated and SOD1 knockdown HeLa cells. Table S3: KEGG pathway enrichment of DEGs in LD100-treated and SOD1 knockdown HeLa cells. Table S4: quality evaluation of transcriptome library in DU145 and RWPE-1 cells. Table S5: GO classification of DEGs in LD100-treated DU145 and RWPE-1 cells. Table S6: KEGG pathway enrichment of DEGs in LD100-treated DU145 and RWPE-1 cells.
