# Supplementary material for: The Specific Inhibition of SOD1 Selectively Promotes Apoptosis of Cancer Cells via Regulation of the ROS Signaling Network
Source: Oxid Med Cell Longev. 2019 Feb 18;2019:9706792. doi: 10.1155/2019/9706792 (PMC6398008; doi:10.1155/2019/9706792)
Supplement: Supplementary Materials — Figure S1: the levels of SOD1 in SOD1 knockdown HeLa cells and key DEGs in LD100-treated HeLa cells. Figure S2: SOD1 activity and H2O2 and O2 •− levels within LD100-treated DU145 and RWPE-1 cells. Figure S3: successful construction of mRNA sequencing in LD100-treated DU145 and RWPE-1 cells. Figure S4: many key genes in representative signaling pathways are selectively regulated upon the specific SOD1 inhibition. Figure S5: the specific SOD1 inhibition selectively regulates cancer pathways in cancer cells. Figure S6: many key genes in the cell cycle and p53 and apoptosis signaling pathways are selectively regulated upon the specific SOD1 inhibition. Figure S7: the specific SOD1 inhibition selectively activates the p53 signaling pathway in cancer cells. Figure S8: specific SOD1 inhibition represses the crosstalk of ERK, PI3K, and NF-κB signaling pathways in cancer cells. Figure S9: the specific SOD1 inhibition increases the intracellular peroxynitrite content. Figure S10: the specific SOD1 inhibition does not affect the growth and proliferation of normal cells. Table S1: quality evaluation of transcriptome library in HeLa cells. Table S2: GO classification of DEGs in LD100-treated and SOD1 knockdown HeLa cells. Table S3: KEGG pathway enrichment of DEGs in LD100-treated and SOD1 knockdown HeLa cells. Table S4: quality evaluation of transcriptome library in DU145 and RWPE-1 cells. Table S5: GO classification of DEGs in LD100-treated DU145 and RWPE-1 cells. Table S6: KEGG pathway enrichment of DEGs in LD100-treated DU145 and RWPE-1 cells. [file 9706792.f1.zip › Revised Supplementary Materials.pdf]

Supplementary Materials

The specific inhibition of SOD1 selectively promotes apoptosis of cancer cells via regulation of the ROS signaling network

Xiang Li[1], Yuanyuan Chen[1], Jidong Zhao, Jiayuan Shi, Mingfang Wang, Shuang Qiu, Yinghui Hu, Yulin Xu, Yanfang Cui, Chunrong Liu[*], Changlin Liu[*]

Key Laboratory of Pesticide and Chemical Biology, Ministry of Education, School of Chemistry, Central China Normal University, Wuhan 430079, Hubei, China

[1]These authors contributed equally to this work.

Email: liuchl@mail.ccnu.edu.cn (Changlin Liu), liucr@mail.ccnu.edu.cn (Chunrong Liu)

Figure S1

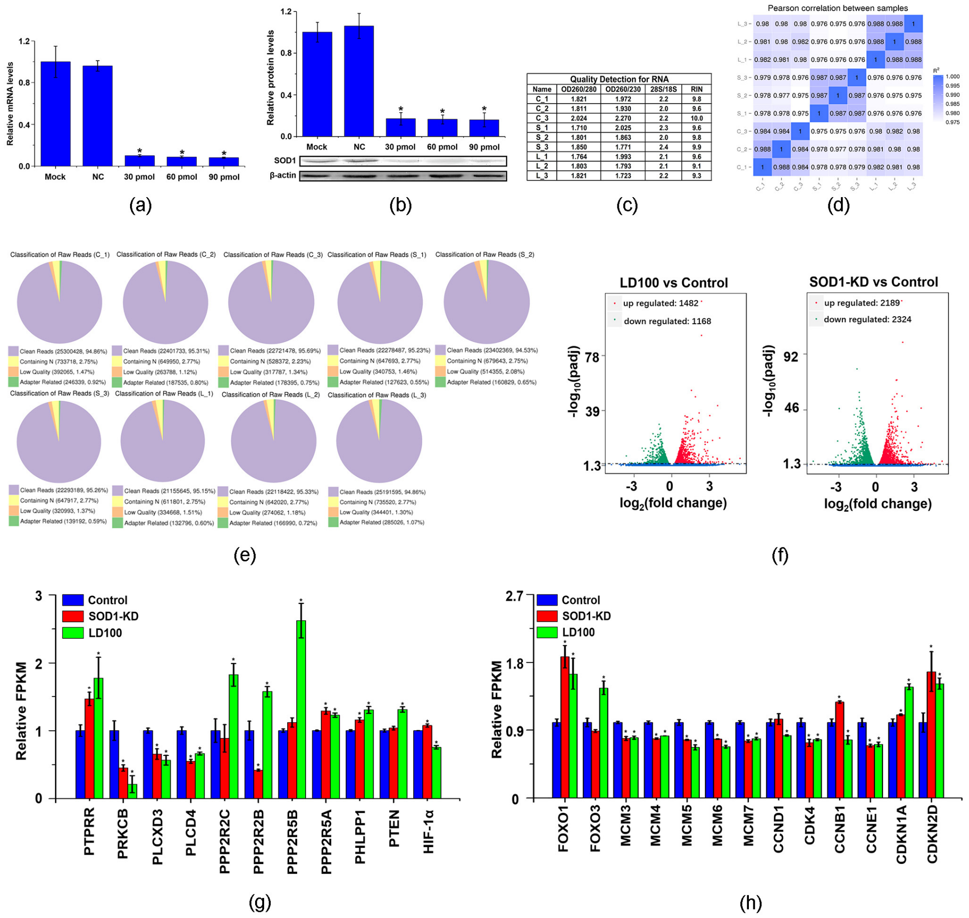

Quality Detection for RNA

Name	OD260/280	OD260/230	28S/18S	RIN
C_1	1.821	1.972	2.2	9.8
C_2	1.811	1.930	2.0	9.6
C_3	2.024	2.270	2.2	10.0
S_1	1.710	2.025	2.3	9.6
S_2	1.801	1.863	2.0	9.8
S_3	1.850	1.771	2.4	9.9
L_1	1.764	1.993	2.1	9.6
L_2	1.803	1.793	2.1	9.1
L_3	1.821	1.723	2.2	9.3

Figure S1: The levels of SOD1 in SOD1 knockdown HeLa cells and key DEGs in LD100-treated HeLa cells, related to Figure 1.

(a, b) After transfection with validated siRNA of SOD1 in HeLa cells, relative mRNA (a) and protein (b) levels of SOD1 are measured by RT-qPCR and western blotting, respectively. The relative intensity of protein bands (means ± SD) in the western blotting is quantified by using ImageJ software and normalized through the negative control, respectively. Representative results from three independent experiments are shown (b). (c) OD260/280, OD260/230, rRNA Ratio (28S/18S) and RNA Integrity Number (RIN) of RNAs extracted from the control-group (designated as C), SOD1 knockdown-group (designated as S) and SOD1 inhibition-group (designated as L) are determined, respectively. (d) Person correlations between parallel mRNA-sequencing samples. The mRNA-sequencing data are representative of three independent experiments. (e) The percentages of clean reads, poly-N containing reads, low quality reads and adapters containing reads in raw data from the mRNA-sequencing samples. (f) The differential mRNA expression displayed as a volcano plot of log2(fold change) vs. the -log10(padj) for each gene. Genes considered significant below padj < 0.05 are highlighted, respectively, in red and green for up-regulation and down-regulation. (g, h) Relative FPKM of key DEGs in LD100-treated HeLa cells. Data are mean of triplicate samples ± SD (*P < 0.05; unpaired Student’s t test; n = 3), and all error bars are SD.

Figure S2

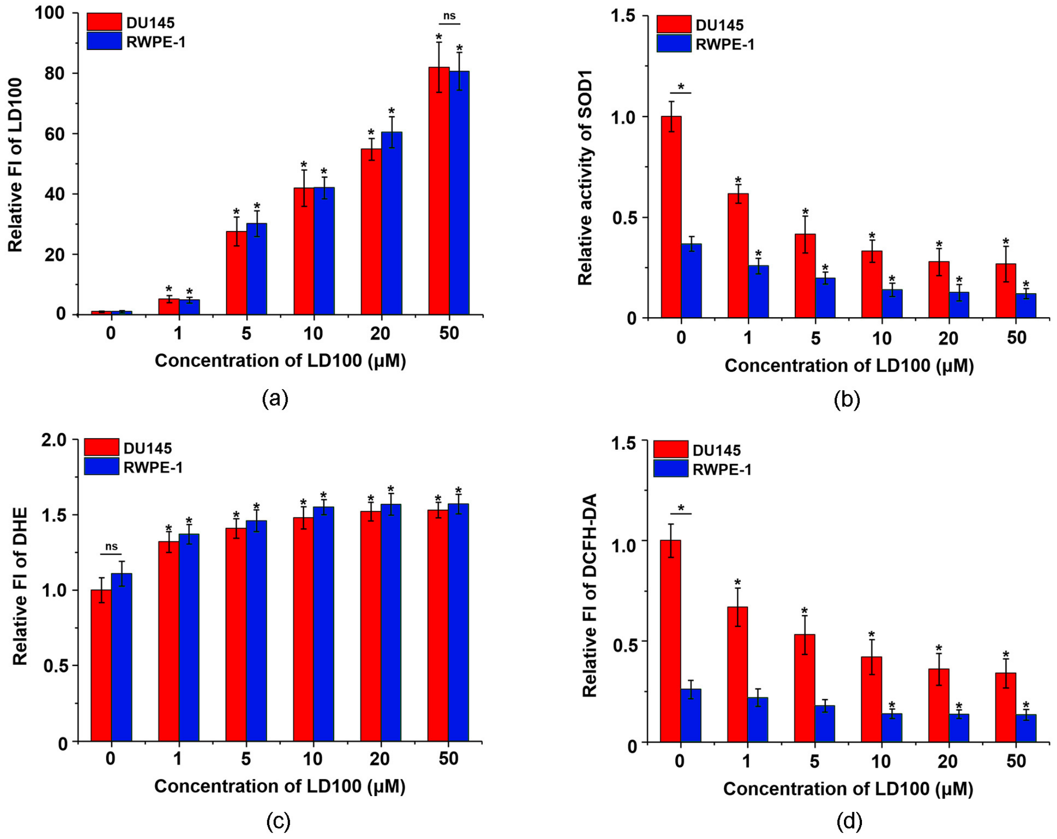

Figure S2: SOD1 activity and H_2O_2 and $O_2^{\bullet-}$ levels within LD100-treated DU145 and RWPE-1 cells, related to Figure 2.

(a) The uptake of LD100 within DU145 and RWPE-1 cells. After treatment with LD100 (0-50 μM) for 24 h, cells were washed, and then the intracellular fluorescence intensity (FI) of LD100 were measured by flow cytometry. (b) After treatment with LD100 (0-50 μM) for 24 h, SOD1 activity within the cells are measured by HT Superoxide Dismutase Assay Kit. (c, d) After treatment with LD100 (0-50 μM) for 24 h, cells are washed and incubated with 5 μM DHE or DCFH-DA in the dark for 20 min. Following subtracting the background fluorescence of LD100, the fluorescence intensity of DHE (c) or DCFH-DA (d) are determined. The DHE and DCFH-DA fluorescence intensity are positively correlated with $O_2^{\bullet-}$ and H_2O_2, respectively. Data are mean of triplicate samples ± SD (ns-no statistically significant; *$P < 0.05$; unpaired Student's t test), and all error bars are SD.

Figure S3

Quality Detection for RNA				
Name	OD260/280	OD260/230	28S/18S	RIN
DC_1	1.947	2.083	2.2	10.0
DC_2	1.968	2.167	2.2	10.0
DC_3	1.992	2.114	2.2	10.0
DL_1	1.950	1.832	2.4	10.0
DL_2	1.976	1.927	2.2	10.0
DL_3	2.015	1.961	2.4	10.0
RC_1	1.967	2.064	2.0	10.0
RC_2	1.970	2.095	2.0	10.0
RC_3	1.959	1.959	2.0	10.0
RL_1	1.967	2.098	2.0	10.0
RL_2	1.955	2.186	1.9	10.0
RL_3	1.952	2.081	1.9	10.0

(a)

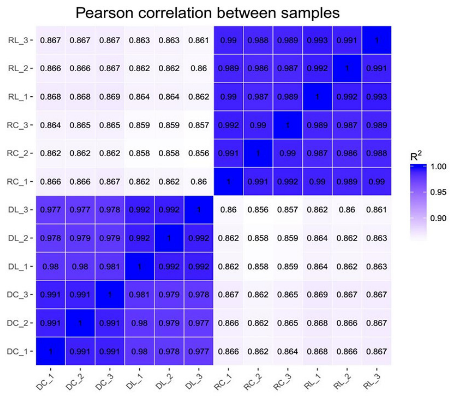

(b)

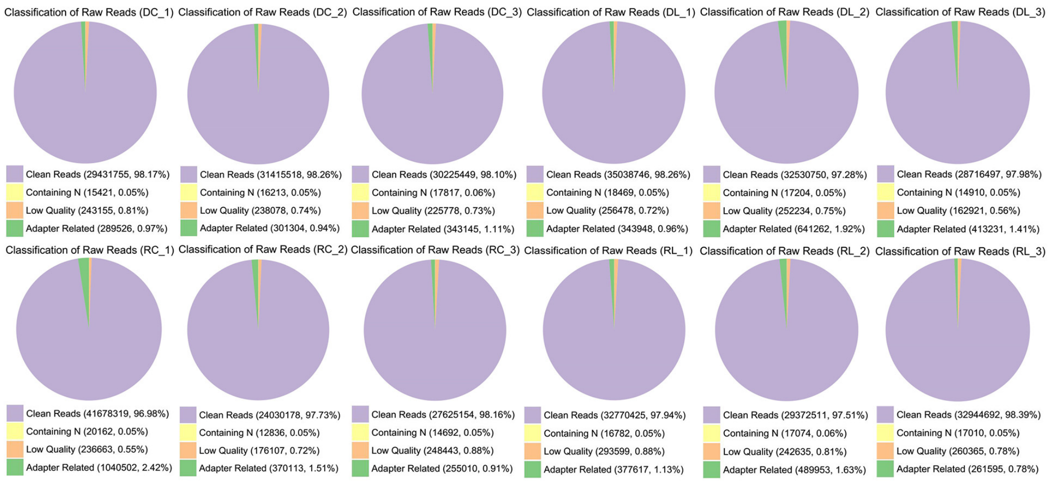

(c)

Figure S3: Successful construction of mRNA-sequencing in LD100-treated DU145 and RWPE-1 cells, relative to Figure 2.

(a) OD260/280, OD260/230, rRNA Ratio (28S/18S) and RNA Integrity Number (RIN) of RNAs extracted from the control-group of DU145 (designated as DC), LD100 treated-group of DU145 (DL), the control-group of RWPE-1 (designated as RC), LD100 treated-group of RWPE-1 (RL) are determined, respectively. (b) Person correlations between mRNA-sequencing samples. The mRNA-sequencing data are the representative of three independent experiments. (c) The percentages of clean reads, poly-N containing reads, low quality reads and adapters containing reads in raw data.

Figure S4

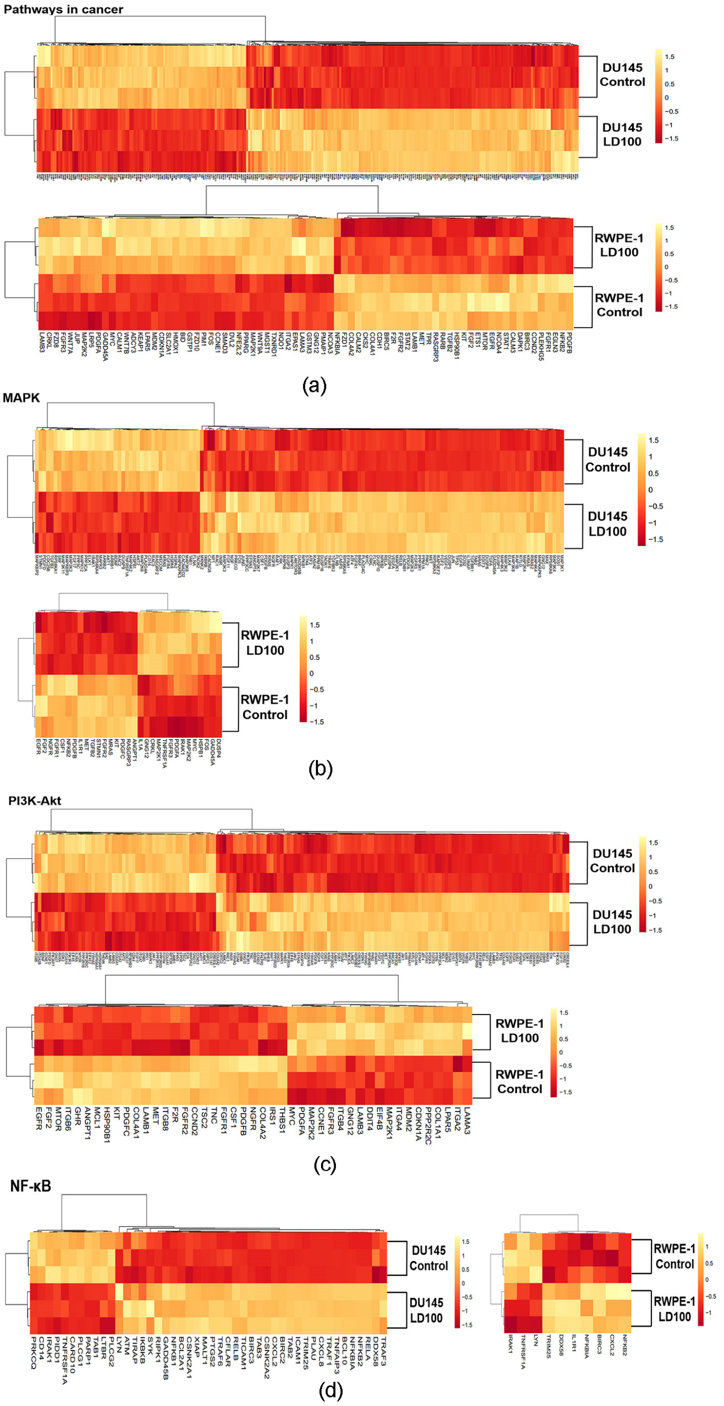

Figure S4: Many key genes in representative signaling pathways are selectively regulated upon the specific SOD1 inhibition, relative to Figure 2.

(a-d) The cluster analysis of DEGs in cancer related signaling pathways such as MAPK, PI3K-Akt and NF-κB of LD100-treated DU145 and RWPE-1 cells. Based on normalized $\log_{10}$(FPKM+1), these heat maps are drawn using yellow and red for high-expressed genes and low-expressed genes, respectively.

Figure S5

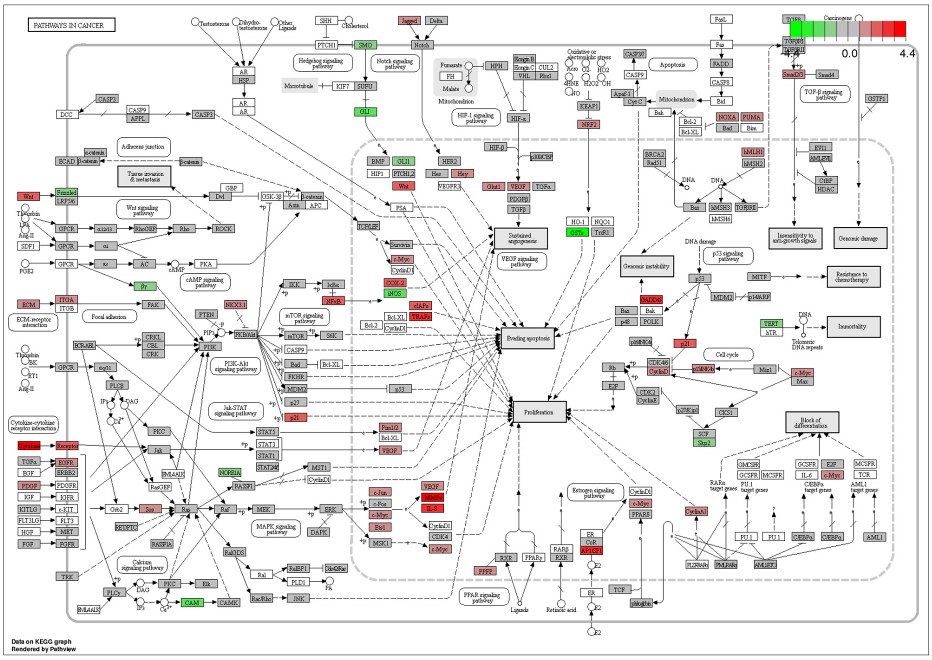

(a)

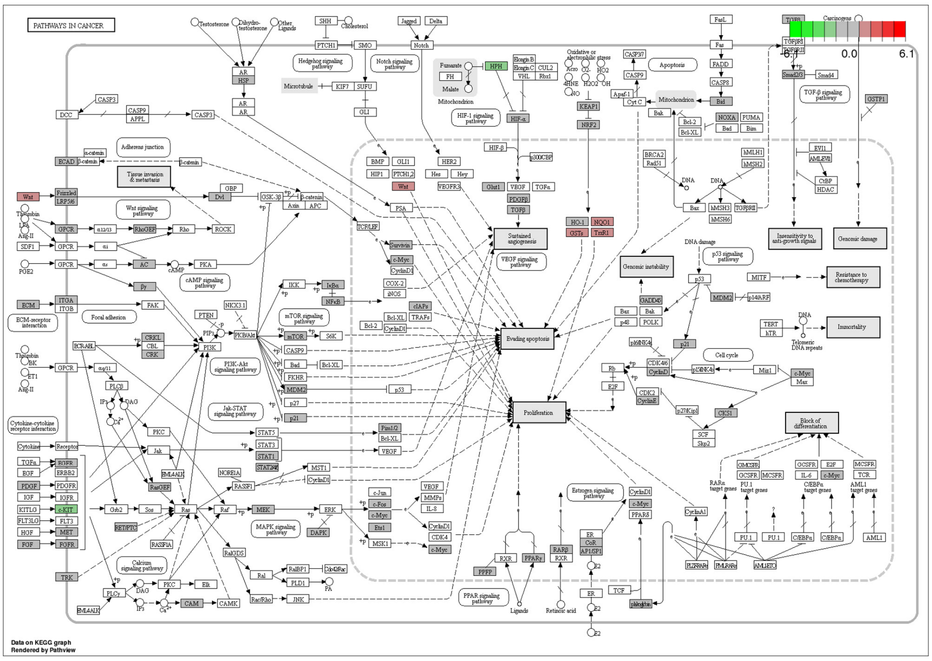

(b)

Figure S5: The specific SOD1 inhibition selectively regulates cancer pathways in cancer cells, relative to Figure 2.

Enriched cancer related KEGG pathways in DU145 and RWPE-1 cells after LD100-treatment, respectively. Based on FPKM, these heat maps are drawn using red and green for high-expressed genes and low-expressed genes, respectively.

Figure S6

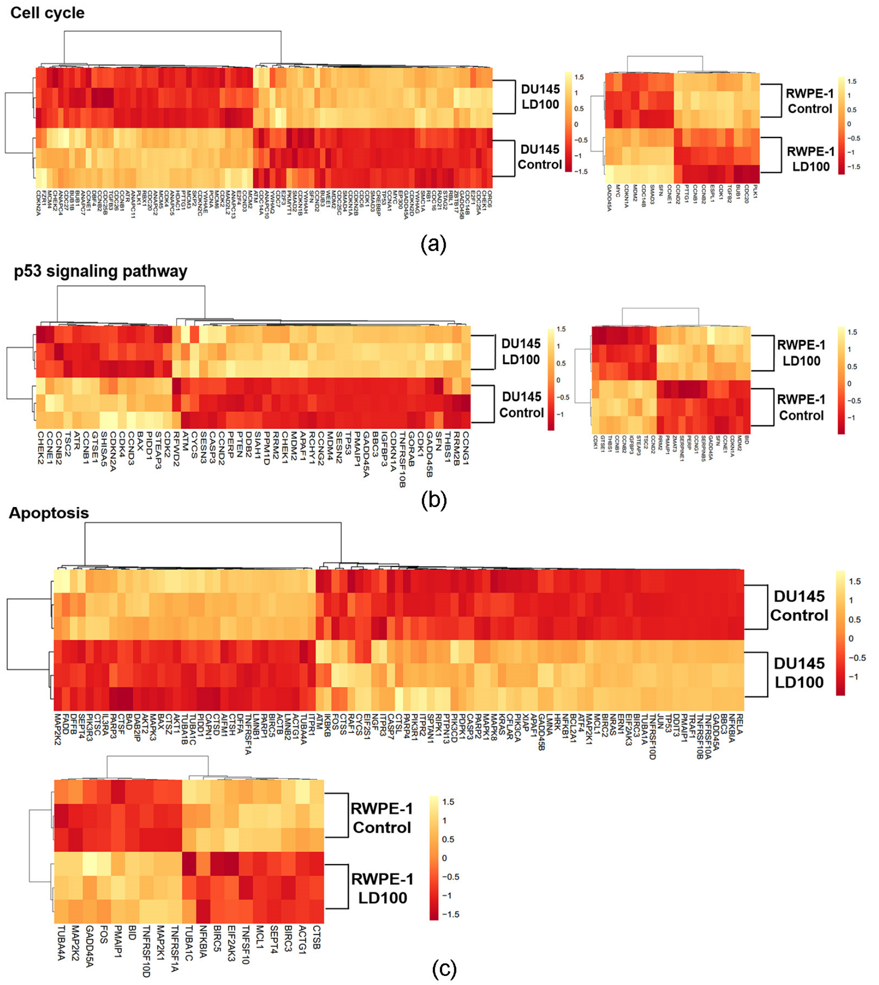

Figure S6: Many key genes in cell cycle, p53 and apoptosis signaling pathways are selectively regulated upon the specific SOD1 inhibition, relative to Figure 2.

(a-c) The cluster analysis of DEGs in cell cycle, p53, apoptosis signaling pathways of LD100-treated DU145 and RWPE-1 cells. Based on normalized $\log_{10}$(FPKM+1), these heat maps are drawn using yellow and red for high-expressed genes and low-expressed genes, respectively.

Figure S7

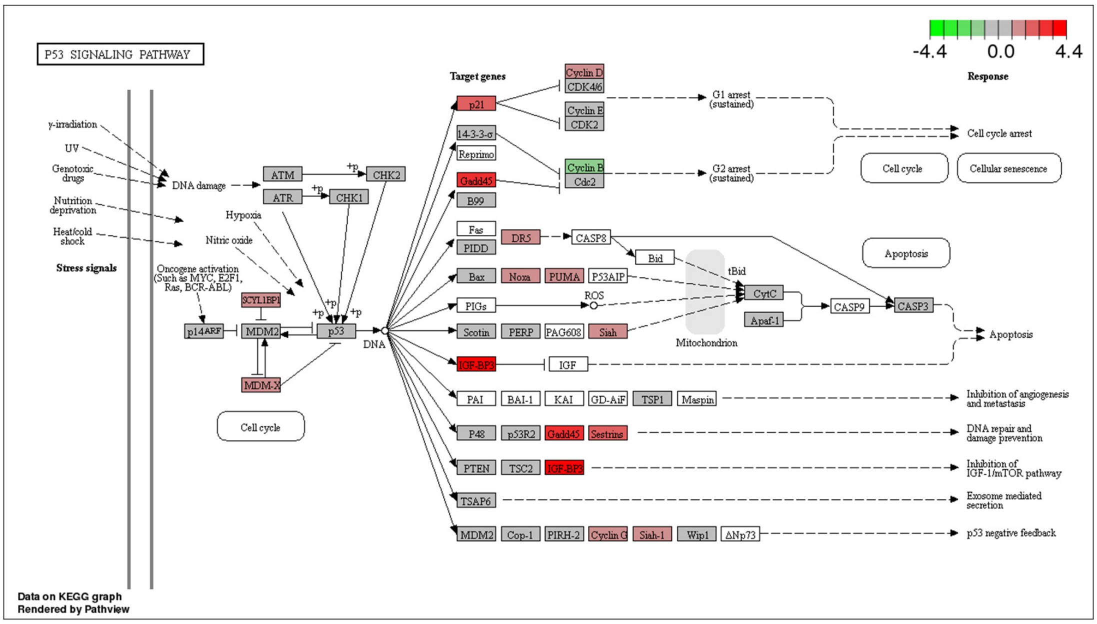

(a)

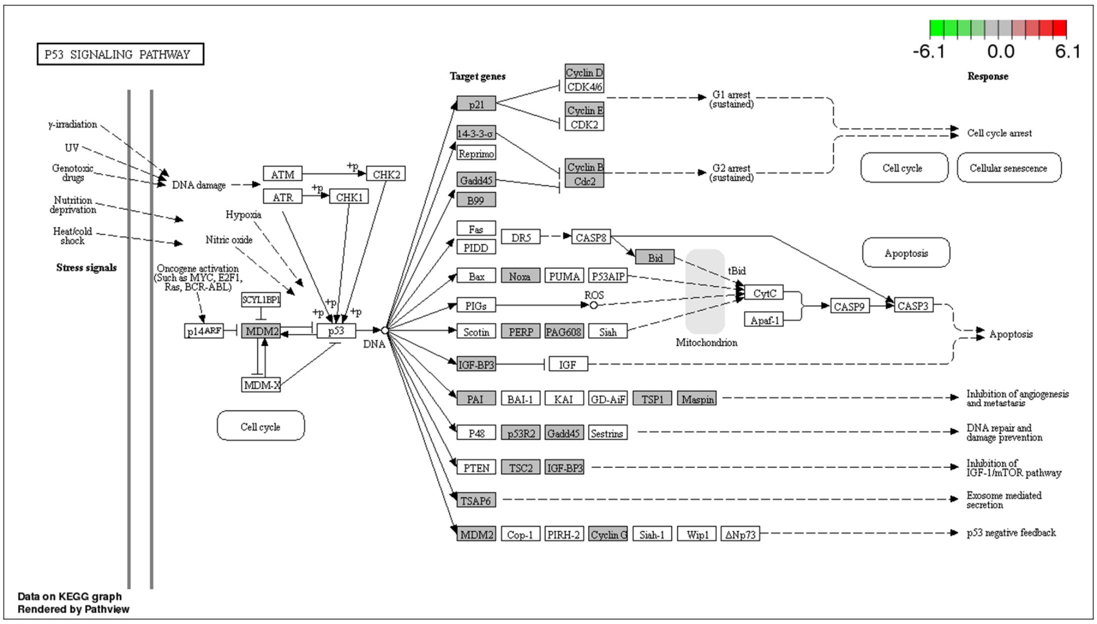

(b)

Figure S7: The specific SOD1 inhibition selectively activates p53 signaling pathway in cancer cells, relative to Figure 2.

Enriched KEGG p53 signaling pathway in LD100-treated DU145 and RWPE-1 cells, respectively. Based on FPKM, these heat maps are drawn using red and green for high-expressed genes and low-expressed genes, respectively.

Figure S8

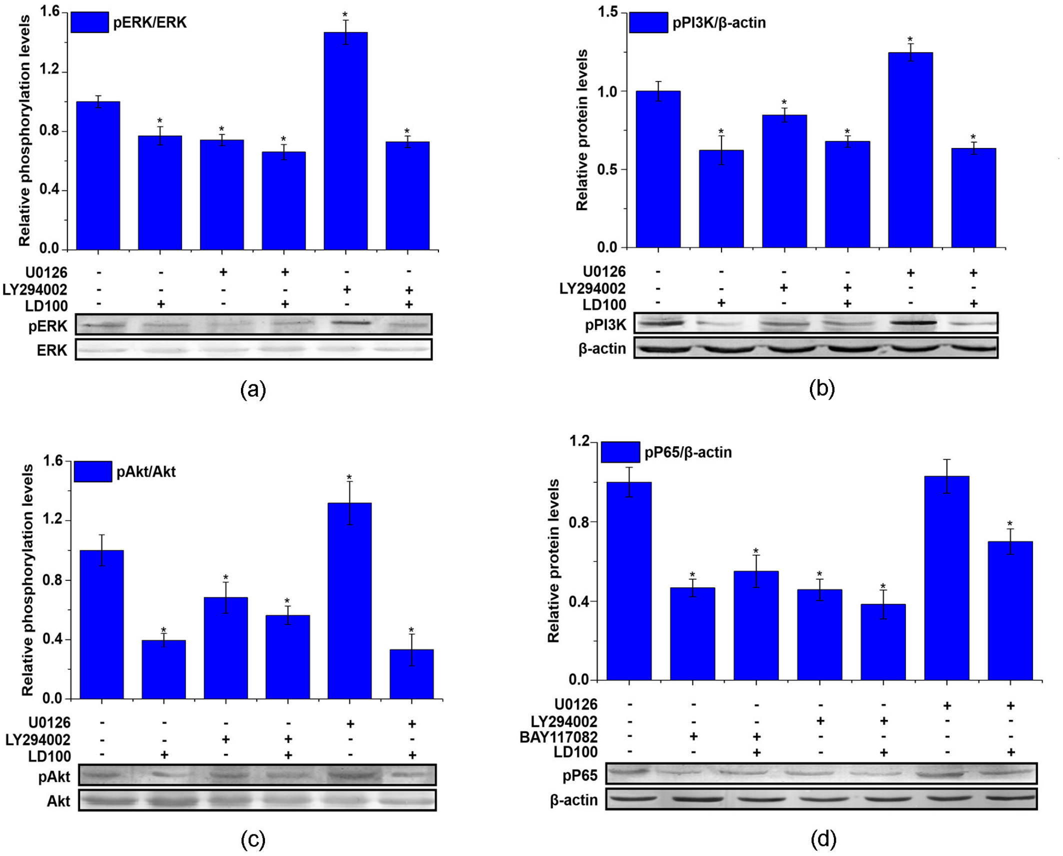

Figure S8: Specific SOD1 inhibition represses the crosstalk of ERK, PI3K and NF-κB signaling pathways in cancer cells.

(a-d) The crosstalk among ERK, PI3K-Akt and NF-κB signaling pathways is evaluated using LD100 and the inhibitors of ERK (U0126), PI3K (LY294002), and NF-κB (BAY117082) by western blotting analysis. HeLa cells are incubated respectively with 50 μM LD100 and inhibitors of these signaling pathways (10 μM U0126, 10 μM LY294002, and 5 μM BAY117082) for 24 h in an orthogonal fashion. The relative intensity of protein bands (means ± SD) in the western blotting is quantified by using ImageJ software and normalized through the negative control, respectively. Representative results from three independent experiments are shown. Data are mean of triplicate samples ± SD (*$P < 0.05$; unpaired Student's t test), and all error bars are SD.

Figure S9

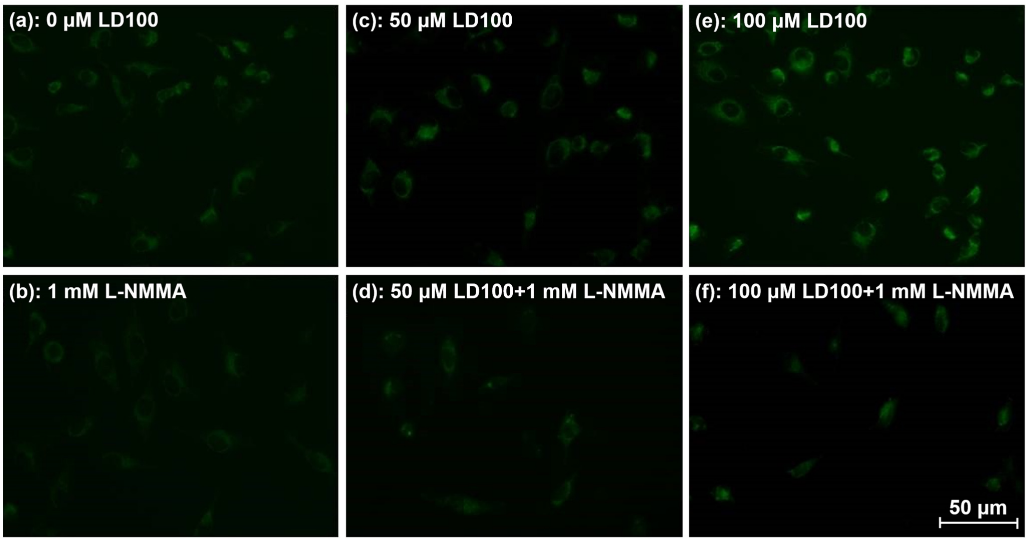

Figure S9: The specific SOD1 inhibition increases the intracellular peroxynitrite content, relative to Figure 5.

(a-f) After incubation for 24 h, respectively, with LD100 (0, 50, 100 μM) and L-NMMA (1 mM), a cell membrane-permeable competitive NOS inhibitor, the level of peroxynitrite in HeLa cells is monitored by Dihydrorhodamine 123 using fluorescence microscopy.

Figure S10

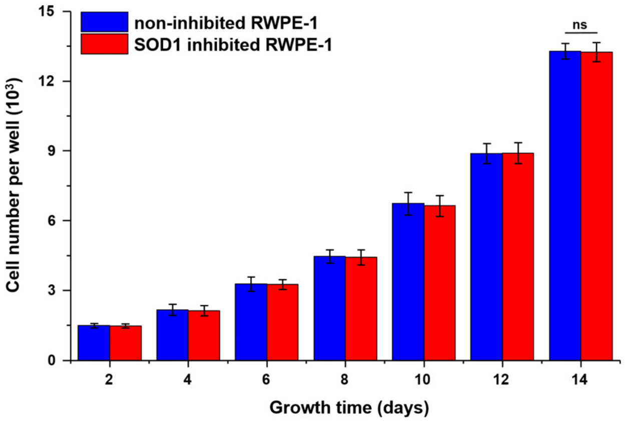

Figure S10: The specific SOD1 inhibition does not affect the growth and proliferation of normal cells, relative to Figure 6.

At first, RWPE-1 cells are counted by flow cytometry. Then, the accurately counted 1.0×10^3 RWPE-1 cells are seeded on a 6-well plate in K-SFM culture medium. After incubating the cells with or without 50 μM LD100 for a different period of time, cell number of each sample is counted by flow cytometry. Data are mean of triplicate samples ± SD (ns-no statistically significant; $^*P < 0.05$; unpaired Student's t test), and all error bars are SD.

Supplemental Table Legends

Table S1: Quality evaluation of transcriptome library in HeLa cells, related to Figure 1.

Table S2: GO classification of DEGs in LD100 treated and SOD1 knockdown HeLa cells, related to Figure 1.

Table S3: KEGG pathway enrichment of DEGs in LD100 treated and SOD1 knockdown HeLa cells, related to Figure 1.

Table S4: Quality evaluation of transcriptome library in DU145 and RWPE-1 cells, related to Figure 2.

Table S5: GO classification of DEGs in LD100 treated DU145 and RWPE-1 cells, related to Figure 2.

Table S6: KEGG pathway enrichment of DEGs in LD100 treated DU145 and RWPE-1 cells, related to Figure 2.
